# Prognostic value of autophagy-related genes based on single-cell RNA-sequencing in colorectal cancer

**DOI:** 10.3389/fgene.2023.1109683

**Published:** 2023-03-30

**Authors:** Yuqi Luo, Xuesong Deng, Weihua Liao, Yiwen Huang, Caijie Lu

**Affiliations:** ^1^ Department of Gastrointestinal and Hepatobiliary Surgery, Shenzhen Longhua District Central Hospital, Shenzhen, Guangdong, China; ^2^ Department of Hepatobiliary Surgery, Shenzhen Second People’s Hospital, The First Affiliated Hospital of Shenzhen University, Shenzhen, Guangdong, China; ^3^ Department of Radiology, Guangzhou Nansha District Maternal and Child Health Hospital, Guangzhou, Guangdong, China; ^4^ Department of Emergency, Nansha Hospital, Guangzhou First People’s Hospital, Guangzhou, Guangdong, China

**Keywords:** colorectal cancer, autophagy related genes, single-cell sequencing, prognostic prediction, immune infiltration, drug sensitivity

## Abstract

**Background:** Colorectal cancer (CRC) is the second most common cancer in China. Autophagy plays an important role in the initiation and development of CRC. Here, we assessed the prognostic value and potential functions of autophagy-related genes (ARGs) using integrated analysis using single-cell RNA sequencing (scRNA-seq) data from the Gene Expression Omnibus (GEO) and RNA sequencing (RNA-seq) data from The Cancer Genome Atlas (TCGA).

**Methods:** We analyzed GEO-scRNA-seq data from GEO using various single-cell technologies, including cell clustering, and identification of differentially expressed genes (DEGs) in different cell types. Additionally, we performed gene set variation analysis (GSVA). The differentially expressed ARGs among different cell types and those between CRC and normal tissues were identified using TCGA-RNA-seq data, and the hub ARGs were screened. Finally, a prognostic model based on the hub ARGs was constructed and validated, and patients with CRC in TCGA datasets were divided into high- and low-risk groups based on their risk-score, and immune cells infiltration and drug sensitivity analyses between the two groups were performed.

**Results:** We obtained single-cell expression profiles of 16,270 cells, and clustered them into seven types of cells. GSVA revealed that the DEGs among the seven types of cells were enriched in many signaling pathways associated with cancer development. We screened 55 differentially expressed ARGs, and identified 11 hub ARGs. Our prognostic model revealed that the 11 hub ARGs including CTSB, ITGA6, and S100A8, had a good predictive ability. Moreover, the immune cell infiltrations in CRC tissues were different between the two groups, and the hub ARGs were significantly correlated with the enrichment of immune cell infiltration. The drug sensitivity analysis revealed that the patients in the two risk groups had difference in their response to anti-cancer drugs.

**Conclusion:** We developed a novel prognostic 11-hub ARG risk model, and these hubs may act as potential therapeutic targets for CRC.

## 1 Introduction

Colorectal cancer (CRC) is the most common malignant tumor of the digestive system in worldwide and ranks second in morbidity and mortality among all cancers in China. According to statistics from China national cancer center, 122,459 patients are newly diagnosed with CRC per year, leading approximately 30,000 deaths yearly (Feng et al., 2019). Owning to advances in treatment and early diagnosis, the outcomes of patients with CRC have improved in recent decades. However, the prognosis of patients with advanced CRC remains poor, with a 5-year survival rate of less than 50%. The most effective method for early diagnosis of CRC is colonoscopy. However, colonoscopy screening for CRC is not widely implemented in China, and approximately 50% of patients with CRC are diagnosed at an advanced staged, leading to a poor prognosis (GBD, 2019 Colorectal Cancer Collaborators, 2022; Sung et al., 2021). Moreover, the initiation and development of CRC are complex, and associated with dysregulated expression of various genes. For example, the expression of *adenomatous polyposis coli* (APC), a tumor suppressor that inhibits the Wnt/β-catenin signaling pathway is downregulated in many patients with CRC (Caspi et al., 2021). Therefore, it is necessary to identify biomarkers for the early diagnosis and targeted treatment of CRC.

Several prognostic biomarkers for CRC have been identified, including non-coding RNA, protein located in different elements of cells, methylated loci in the promoter of oncogene or tumor suppressor genes, and mutated drivers (Chen et al., 2021; Martelli et al., 2022). These biomarkers are associated with the altered the phenotypes of CRC cells and play a role in the development and progression of CRC. For example, high expression of the hominid-restricted retrogene POU5F1B in tumor tissues is associated with a poor outcome of patients with CRC and promoted the cancer growth and metastasis by activating intracellular signaling events and releasing trans-acting factors (Simo-Riudalbas et al., 2022). Nowadays, with the deepening of research, recently report has found autophagy proteins have prognostic value for CRC (Hu et al., 2022). Autophagy is a vital physiological process that plays an important role in maintaining cellular homeostasis through lysosome-dependent cellular degradation (Chen et al., 2021). It is regulated by several genes termed autophagy-related genes (ARGs). Autophagy critically has been reported affecting the carcinogenesis of CRC (Huang et al., 2018; Gao et al., 2022). For example, Sphk1-driven autophagy enhanced CRC metastasis (Wu et al., 2021). However, bivalent β-Carbolines induced autophagy to depressed CRC growth (Zhang et al., 2021), which means autophagy played a balance role in the occurrence and death of tumors. Therefore, identifying the hub ARGs in CRC can deepen our understanding of the genomic alteration between CRC and normal tissues. Moreover, hub ARGs may act as prognostic biomarkers and therapeutic targets for CRC.

Owing to recent progress in single-cell RNA sequencing (scRNA-seq), CRC cells can be classified into different subtypes using scRNA-seq and screen the differentially expressed genes (DEGs) among the different cell types, which can deepen our understanding of the heterogeneity of intratumoral (within tumor cells) and intertumoral (within the tumor microenvironment [TME]) heterogeneity (Casasent et al., 2018; Zheng et al., 2018). In addition, scRNA-seq can detect circulating tumor cells and can be used for assessing the risks of metastasis in clinical settings (Beshnova et al., 2020). However, there are only a few reports on the application of scRNA-seq to determine and validate the prognostic signature of ARGs in CRC, and previous preliminarily studies validating the prognostic value of ARGs in CRC were performed using bulk transcriptomic profiles (Huang et al., 2018; Gao et al., 2022).

This study aimed to identify prognostic AGRs biomarkers among subpopulations of CRC tissue using single-cell profiling. We downloaded scRNA-seq data of CRC from the Gene Expression Omnibus (GEO) and obtained RNA-sequencing (RNA-seq) data together with clinical information from The Cancer Genome Atlas (TCGA). Additionally, we screened hub ARGs and constructed a prognostic model based on their expression levels and clinical factors. Finally, we validated the predictive value of ARGs for patients with CRC to investigate their possible role in individualized treatments.

## 2 Methods

**FIGURE 1:**
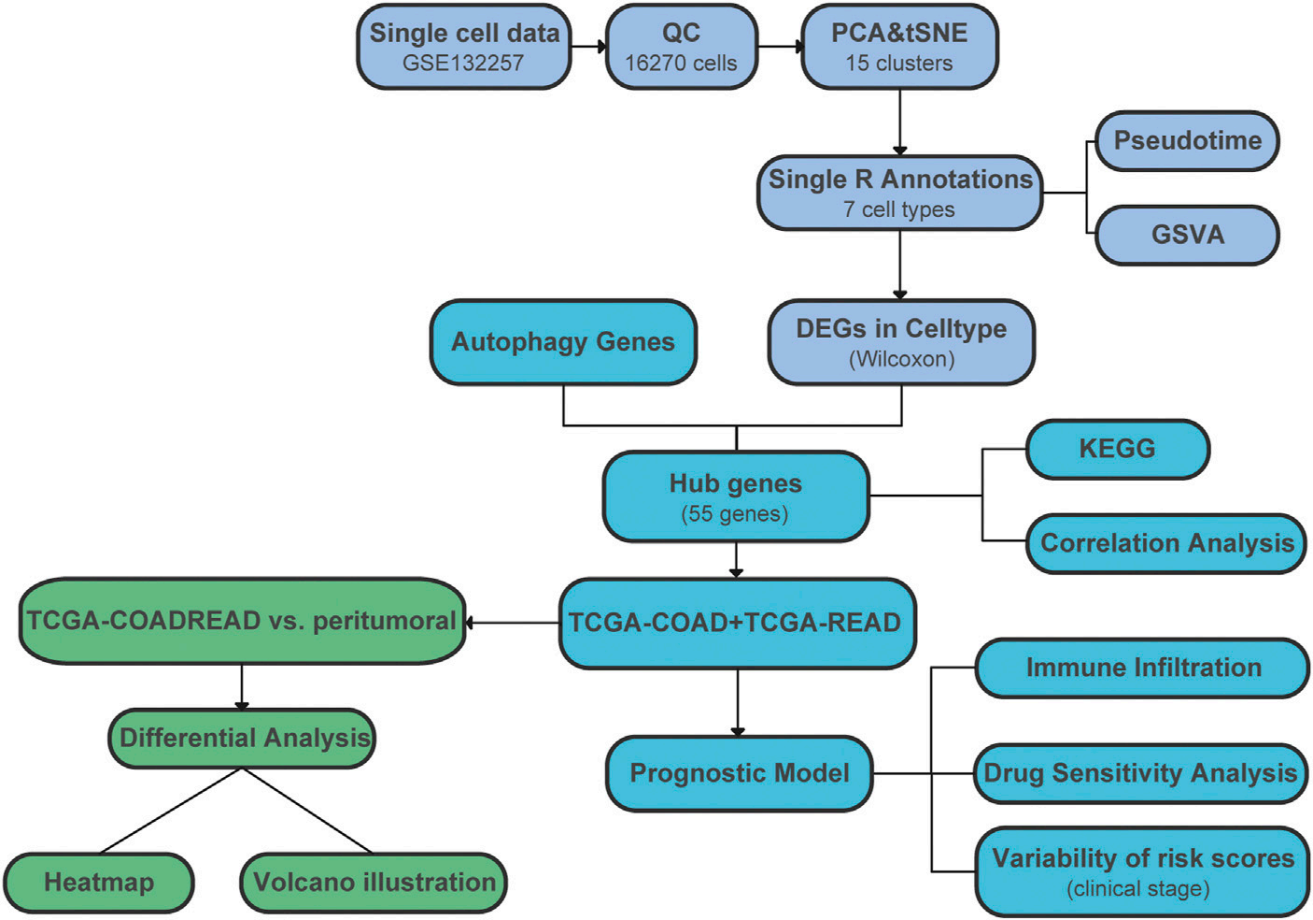
Flow chart of analyses performed in this study.

The workflow of data processing and analysis is outlined in Figure 1.

### 2.1 Acquisition of cell samples and CRC population cohorts

The scRNA-seq dataset GSE132257 (five CRC tissues and five normal tissues, platform: GPL16791) was downloaded from GEO (https://www.ncbi.nlm.nih.gov/geo/) (Lee H. O. et al., 2020). The RNA-seq data (level 3: raw counts) of colon adenocarcinoma (COAD) and rectum adenocarcinoma (READ) were downloaded from TCGA portal (https://portal.gdc.cancer.gov/) using the R package TCGAbiolinks (v 2.22.4) (Colaprico et al., 2016) and then merged into the expression matrix. Next, Raw counts were converted to fragments per kilobase per million (FPKM) using the R package DESeq2 based on gene lengths. A total of 417 CRC samples with survival information were used for prognostic analysis.

### 2.2 Quality control and processing of single-cell data from CRC tissues

The R package Seurat (v4.0.5) was used for quality control (Satija et al., 2015). A Seurat object was created for the expression matrix GSE132257. The proportion of mitochondria genes reflect the homeostasis of cells, and a higher proportion of mitochondrial genes than other genes in cells indicates that the cells are in a state of stress. Therefore, we restricted the proportion of mitochondria genes to less than 20%. Similarly, we excluded the cells with genes numbers less than 200 or more than 3,000, which represented low-quality cells or non-single cells respectively. Finally, there were 16,270 cells were obtained after quality control.

Cells were then normalized for sequencing depth using the NormalizeData function, and the standardized method was default “LogNormalize”; the top 2,000 variable genes were detected using the variance-stabilizing transformation (vst) method in the “FindVariableFeatures” function. Next, principal component analysis (PCA) was used for identifying significant principal components (PC), and *p*-value distributions were visualized using the “Elbowplot” function. We selected 32 PCs for t-distributed stochastic neighbor Embedding (tSNE) analysis, and k-nearest neighbors based on Euclidean distance were constructed in PCA spaces, where the clusters were then identified using the Louvain algorithm. Finally, the cells were divided into 15 clusters with a resolution of 0.2. In addition, the “RunTSNE” function was used for dimensional reduction, to visualize and explore the dataset.

We used “BlueprintEncodeData” as a reference dataset in the R package SingleR (Aran et al., 2019) to annotate the clusters and cell categories, and identified seven cell types, which were T-cells, B-cells, epithelial cells, myeloid cells, fibroblasts, mast cells and endothelial cells. To screen the DEGs among the seven cell types, we performed Wilcoxon rank-sum test using the “FindAllMarkers” function.

### 2.3 Single-cell trajectories reconstruction and analysis

Cell trajectories were inferred using the default parameters of the R package Monocle (Qiu et al., 2017). First, the gene expression matrices from Seurat were exported to Monocle and cell datasets were constructed. Next, we defined variable genes using the “dispersionTable” function and then sorted the cells using the “setOrderingFilte” function. Finally, the “DDRTree” method was used for dimensional reduction andthe position of cells along the trajectory of cell states was determined using the “orderCells” function. A cell differentiation time tract was formed based on cluster characteristics and marker gene analysis.

### 2.4 Gene set variation analysis (GSVA)

The R package “GSVA” is a non-parametric and unsupervised method for estimating the variation in gene set enrichment in gene expression data (Hanzelmann et al., 2013). ScRNA-seq data of CRC tissues were used as input for the GSVA algorithm. We downloaded c2.cp.kegg.v7.5.1.symbols.gmt and c7.immunesigdb.v7.5.1.symbols.gmt respectively from the Molecular Signatures Database (MSigDB, https://www.gsea-msigdb.org/gsea/msigdb/) (Liberzon et al., 2015) as reference gene sets. The enrichment score (ES) of each pathway in the gene sets was then calculated. The differential ES of pathways among different cell clusters was determined using the R package “limma” (v 3.50.0) (Ritchie et al., 2015), and the top three pathways (selected based on the t-value) of each cell cluster were used for plotting heatmaps. Statistical significance was set at *p* < 0.05.

### 2.5 Differential expression of ARGs

First, we analyzed the differential expression of ARGs among the seven cell types. We obtained 489 ARGs from the human autophagy database (HADb, http://www.autophagy.lu/index.html) and Human Autophagy Moderator Database (HAMdb, http://hamdb.scbdd.com/home/download/) (Deng et al., 2018). We identified the intersection between the 489 ARGs and the DEGs of the seven cell types to determine the differentially expressed ARGs.

Next, we screened the DEGs between CRC tissues and normal tissues based on TCGA-RNA-seq data. The R package DESeq2 (v1.34.0) (Love et al., 2014) was used to identify DEGs, using the threshold parametric *p* < 0.05 and |logFC| > 1. Finally, the differential expression ARGs between CRC tissues and normal tissues were selected, and clustering heatmaps and volcano plots were plotted using the ggplot2 package.

### 2.6 Cell culture

The human normal colon epithelial cells (NCM460D) and CRC cell (SW480) were purchased from the Chinese Academy of Sciences (Shanghai, China). The NCM460D cells were cultured in Dulbecco’s Modified Eagle Medium medium (Gibco) with 10% fetal bovine serum (FBS) and 1% streptomycin/penicillin. The SW480 cells were cultured in L15 base medium with 10% FPS and 1% streptomycin/penicillin. All the cell lines were cultured in an incubator with 37°C and 5% CO_2_.

### 2.7 qRT-PCR

According to the manufacturer’s instructions, TRIzol reagent (Invitrogen) was applied to extract total RNA from the cells. Next, PrimeScript RT Master Mix Kit (TaKaRa) was applied to reverse transcribe RNA to cDNA. qRT-PCR was performed using an ABI StepOnePlus Real-time PCR system (Applied Biosystems) with SYBR Green Real-time PCR Master Mix (Vazyme). The house-keeping gene, GAPDH was used to normalize the mRNA. The primers were designed in Supplementary Table S1.

### 2.8 siRNA knockdown and transfection

The cells were spread on six-well plates. Next, cell transfection was performed with LipofectamineTM 2000 (Invitrogen). After 6 h, complete medium was added to the cells. RiboBio (Guangzhou, China) synthesized the small interfering RNA (siRNA) sequences required for this study.

### 2.9 Western blot

RIPA lysis buffer (Thermo Fisher) was applied to extract proteins from cells. Western blot assay was performed with reference to previous report (He et al., 2021). The antibodies used were: PDK4 (1:7000; Proteintech, 12949-1-AP), GAPDH (1:15000; Proteintech, 60004-1-Ig), Goat Anti-Mouse IgG H&L(HRP) (1:1000; Beyotime, A0216), Goat Anti-Rabbit IgG H&L(HRP) (1:1000; Beyotime, A208). Western blots were detected by ECL luminescence kit (Thermo Fisher) and visualized by chemiluminescence apparatus. ImageJ software was used for quantification.

### 2.10 Cell proliferation assay

SW480 was seeded into 96-well plates at a density of 1 × 10^3^ cells/well. At different time points, 10 μL CCK-8 solution was added. After 2 h, the OD values were measured at 450 nm using a microplate reader.

### 2.11 Transwell assay

For migration assays, 1×10^5^/mL SW480 were seeded in the upper chamber of a 24-well plate containing 0.4 mL of serum-free medium. 0.6 mL of culture medium containing 10% FBS was added to the lower chamber. After 24 h, cells were sequentially fixed with methanol for 15 min and stained with 0.1% crystal violet for 30 min. For invasion assays, the upper chamber was coated with Matrigel and then seeded with SW480. Medium containing 10% FBS was added to the lower chamber. Cell migration and invasion were observed under a microscope.

### 2.12 Correlation and enrichment analyses of the differentially expressed ARGs among different cell types

The correlation coefficient of differentially expressed ARGs among different cell types was obtained using the R package corrr, and the network graph was plotted based on correlations using the “network plot” function. The Kyoto Encyclopedia of Genes and Genomes (KEGG) is a large database containing genomic, biological processes, diseases, and drug information (Kanehisa et al., 2017). We analyzed the KEGG pathway enrichment of differentially expressed ARGs using the R package “clusterProfiler” (v4.2.0) (Yu et al., 2012), and visualized it using a lollipop diagram. Statistical significance was set at *p* < 0.05.

### 2.13 Construction and assessment of prognostic model

The fragments per kilobase of transcript per million mapped reads (FPKM) values of 417 CRC samples with survival information were used construct a prognostic model. First, we performed a survival analysis for the differentially expressed ARGs among the seven cell types, determined the ARGs that were significantly associated with the overall survival (OS) of patients with CRC (*p* < 0.05), and then selected the important genes according to the Boruta feature. Next, we divided the samples from TCGA database into two groups at ratios of 7:3, one of which was the training set for the prognostic model and the other was the validation set for the prognostic model. We constructed a prognostic model according to a multivariate Cox regression analysis and calculated the prognostic risk score of each patient using the following formula:

$$riskScore=\underset{i}{\sum }Coefficient\;\left(gene_{i}\right)\;*Expression\;\left(gene_{i}\right)$$

Based on the median of risk score, 417 patients were assigned to the high- or low-risk groups. Kaplan–Meier survival curves were then calculated to compare the OS between the high- and low-risk groups. In addition, to evaluate the predictive accuracy and sensitivity of our prognostic model, time-dependent receiver operating characteristics (ROC) curves of the 1-, 3- and 5- year were constructed. Next, we compared the difference in risk score according to age, sex, and TNM stage using the *t*-test. Statistical significance was set at *p* < 0.05.

### 2.14 Immune infiltration analysis

The absolute abundance of immune- and non-immune-stromal cell populations was estimated according to RNA-seq of CRC tissues from TCGA using the CIBERSORT method (Chen et al., 2018) to assess the proportion of 22 types of immune cell subsets: memory B cells, naive B cells, activated dendritic cells, resting dendritic cells, Eosinophils, M0 macrophages, M1 macrophages, M2 macrophages, activated mast cells, resting mast cells, monocytes, neutrophils, activated NK cells, resting NK cells, plasma cells, activated memory CD4^+^ T cells, resting memory CD4^+^ T cells, naive CD4^+^ T cells, CD8^+^ T cells, follicular helper T cells, gamma delta T cells and regulatory T cells (Tregs). For every sample, the total immune score of immune cell subsets was 1, and the immune cell abundance between the high- and low-risk groups was compared using the *t*-test (*p* < 0.05).

### 2.15 Prediction of drug sensitivity

The R package pRRophetic (v0.5) (Geeleher et al., 2014) was used to predict drug sensitivity. The half-maximum inhibitory concentration (IC_50_) of each patient was assessed by rigid regression according to the cancer genome project (CGP) database, and the prediction accuracy was estimated by 10-fold cross-validation. The difference in drug sensitivity between the high-risk and low-risk groups was determined by the *t*-test. Statistical significance was set at *p* < 0.001.

### 2.16 Statistical analyses

Data were expressed as the mean +standard deviation (SD). Statistical analyses were performed using the R software (https://www.r-project.org/, v4.1) and GraphPad Prism 8.0. Comparison of continuous variables between the two groups was performed by independent Student’s t-test, multiple groups (≥3) was One-way ANOVA with Tukey’s *post hoc* test, and non-normally distributed data were analyzed using the Mann-Whitney *U* test or Wilcoxon signed-rank test. The Chi-squared test or a Fisher’s exact test was used to compare the categorical variables. Correlation analysis was performed by calculating Pearson correlation coefficients. Statistical significance was set at *p* < 0.05 or *p* < 0.01.

## 3 Results

### 3.1 Cellular heterogeneity and reconstruction of cell trajectory

**FIGURE 2:**
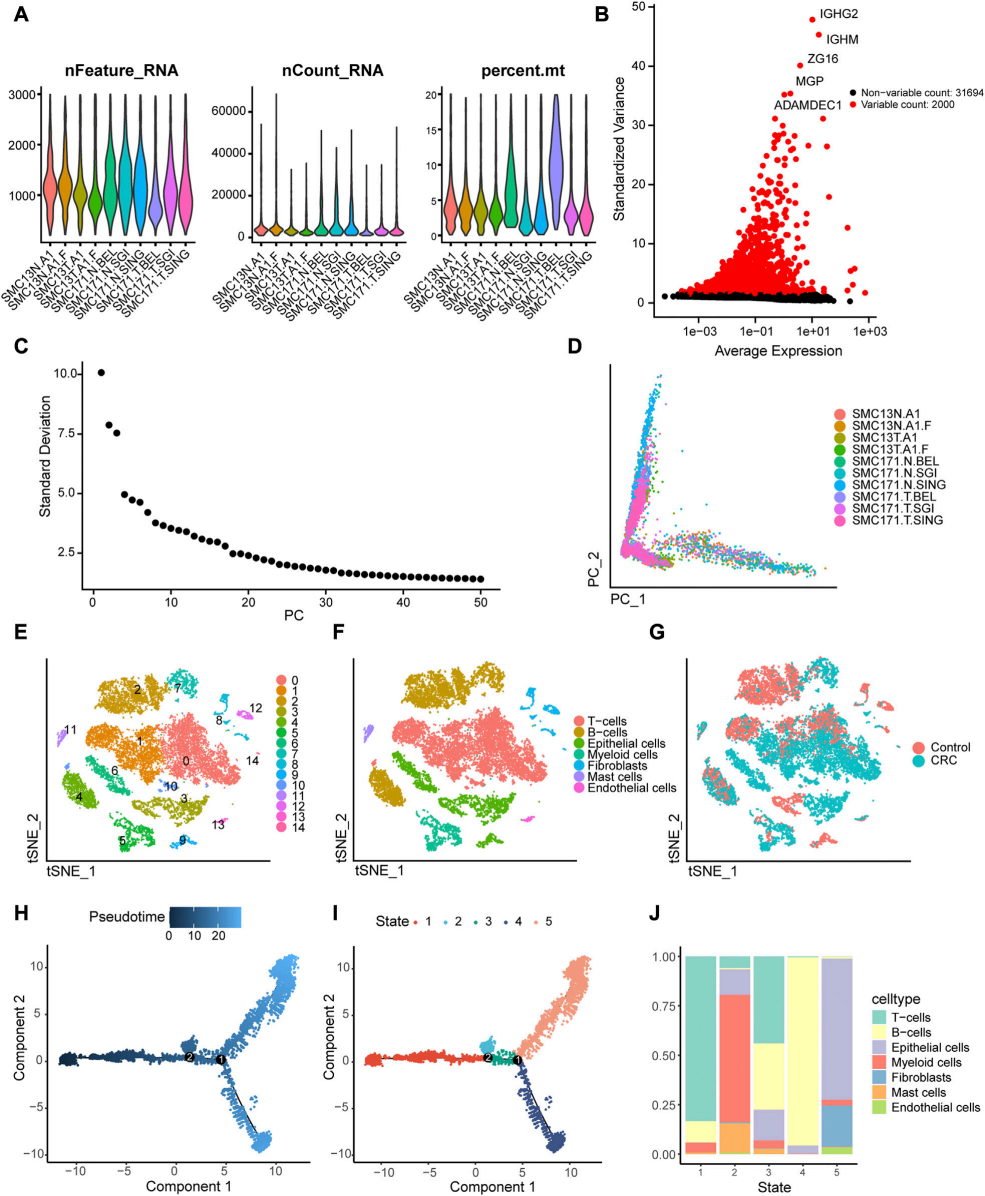
Identification of 15 cell clusters based on scRNA-seq. **(A)** Quality control of scRNA-seq for 10 samples from the GSE132257 dataset. A total of16, 270 cells were included for analysis. **(B)** Scatter plot displaying the hypervariable genes in cells. The top five genes are indicated. **(C)** Elbow plot of the optimal number of principal components (PC) for further analysis. **(D)** Distribution of cells in the principal component analysis (PCA). **(E)**Cluster analysis of scRNA-seq. **(F)** Annotation the cell subpopulations using singleR package. **(G)** Distribution of the cells between colorectal cancer (CRC) and normal tissues. **(I, J)** Single-cell trajectories reconstruction reveals five distinct Monocle stages, and pseudotime plot is colored based on pseudo-time **(H)** and Monocle stages **(I)**. **(J)** Proportions of different types of cells in the five Monocle stages.

Based on quality control and normalization of scRNA-seq data, we obtained 16,270 cells after filtering (Figure 2A). We selected 2,000 high-variability genes for subsequent analysis, and marked the top five genes in Figure 2B. Next, we used PCA method to screen significantly correlated genes, and 32 PCs were then selected for further analysis (Figure 2C), and the cell distributions in PCA were visualized using “Dimplot” function (Figure 2D). We assigned the 16,270 cells into 15 independent clusters using the visualization of tSNE dimension reduction (Figure 2E) and annotated the clusters using the R package SingleR. As shown in Figure 2F, cluster 0, 1, and 10 were annotated as T cells (7,334 cells, 45.08%); clusters 2, 4, and 7 were annotated as B cells (9,518 cells, 40.97%); clusters 3, 6, and 9 were annotated as epithelial cells (2,690 cells, 16.53%); cluster 5 was annotated as myeloid cells (1,041 cells, 6.40%); cluster 8, 12, and 14 were annotated as fibroblasts (706 cells, 4.34%); cluster 11 was annotated as mast cells (207 cells, 1.27%); and cluster 13 was annotated as endothelial cells (113 cells, 0.69%). Similarly, we visualized the distribution of the seven cell types in CRC and normal tissues using tSNE (Figure 2G). Finally, we performed pseudotime analysis for scRNA-seq data and mapped the cells to the pseudotime trajectory. We colored the trajectorial map based on peseudotime (Figure 2H) and stage (Figure 2I), and then displayed the proportion of different cell types in the five stages of the cell trajectory. As shown in Figure 2J, T cells, B cells and epithelial cells were mainly distributed in stage 1, 4, and 5, respectively.

### 3.2 Pathway enrichment analysis across different cell types

**FIGURE 3:**
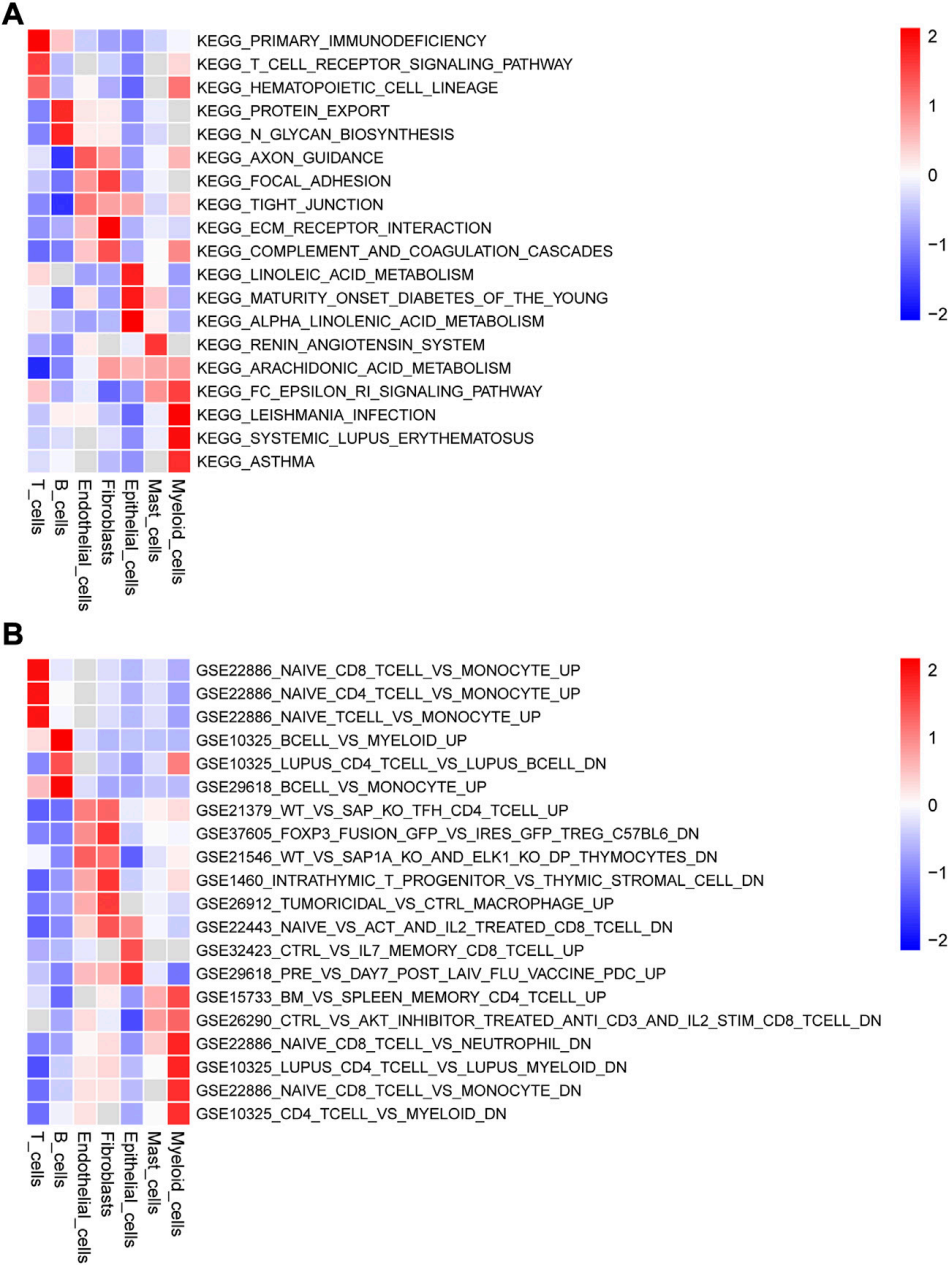
Gene set variation analysis (GSVA) of the significantly enriched signaling pathways (Top 3) among the seven types cells using **(A)** c2.cp.kegg.v7.5.1.symbols.gmt and **(B)** c7.immunesigdb.v7.5.1.symbols.gmt as reference gene sets.

We obtained the enrichment score of each pathway for each cell type and identified the pathways with significant differences in enrichment score. The top three pathways of the seven cell types are visualized as heatmaps in Figure 3. Several KEGG pathways involved in the initiation and development of cancer were enriched in some cell types (Figure 3A). For example, focal adhesion, tight junction, and ECM receptor interaction, which were enriched in endothelial cells, are associate with the migration and invasion of tumor cells, whereas protein export and N-glycan biosynthesis, which were enriched in B cells, are related to the substance metabolism. Figure 3B shows the pathways associated with the cell state within the immune system; many pathways related to the immune response were enriched in the seven cell types.

### 3.3 Differential expression of ARGs

**FIGURE 4:**
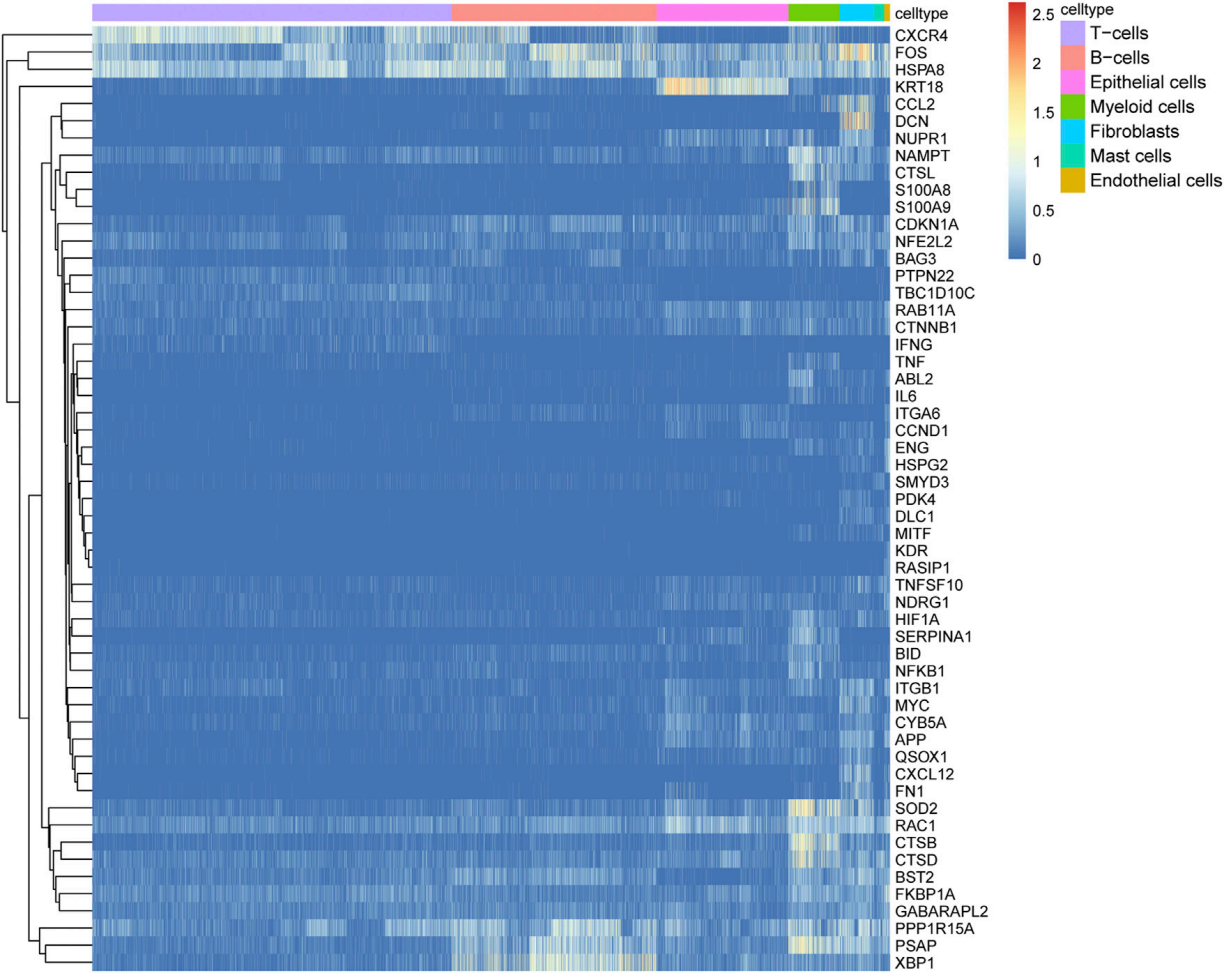
Expression heatmap of 55 autophagy-related genes (ARGs) among the seven cell types. Blue-red intensities represent the expression levels of ARGs from low (blue) to high (red).

To identify differentially expressed ARGs, we considered the intersection of DEGs among the seven cell types and ARGs, which revealed 55 differentially expressed ARGs. The expression levels of the 55 ARGs in the seven cell types are displayed in a heatmap. As shown in Figure 4, CXCR4, FOS, and HSPA8 were highly expressed in B cells; KRT18 was highly in epithelial cells; SOD2, RAC1, CTSB, and CTSD were overexpressed in myeloid cells.

**FIGURE 5:**
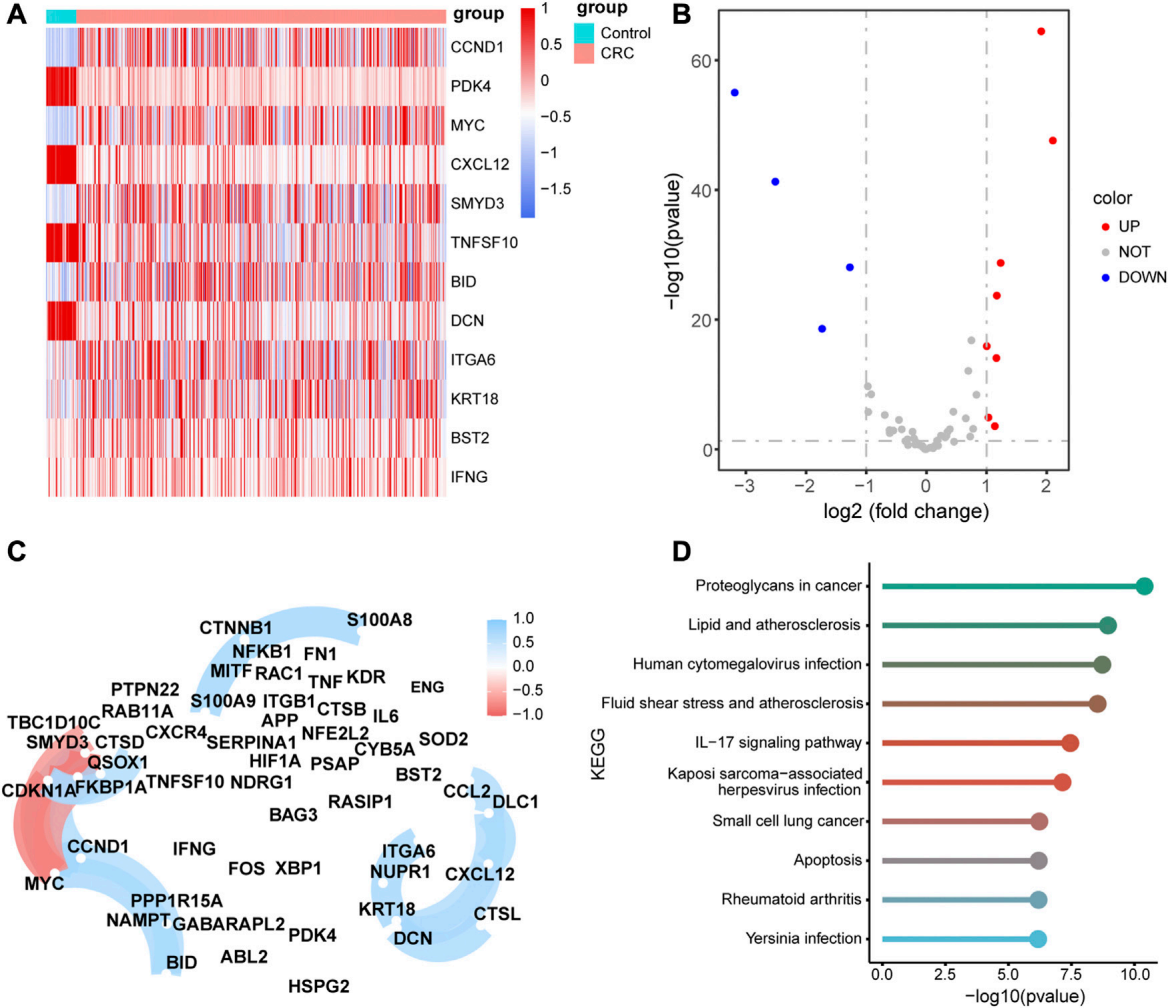
Differential, correlation, and signal pathway enrichment analysis of the 55 autophagy-related genes (ARGs) **(A)** Heatmap of the differential expression levels of11 ARGs expression levels between colorectal cancer (CRC) and normal adjacent tissues. **(B)** Volcanic plot of the differential expression ARGs between CRC and normal adjacent tissues. **(C)**Correlation network diagram of the 55 ARGs. The red color represents a positive correlation and blue color represents a negative correlation. **(D)** Top 10 enriched metabolic pathway of the 55 ARGs.

Next, we compared the expression levels of 55 ARGs between CRC tissues and adjacent normal tissues based on the TCGA-RNA-seq data. Eight ARGs were significantly upregulated in CRC tissues, whereas four ARGs were significantly downregulated compared to adjacent normal tissues (Figures 5A, B, and Supplementary Table S2). To verify the biological function of these ARGs in colon cells, we chose the 8 ARGs were selected for validation. The results showed that PDK4, TNFSF10, CCDN1, and BID was highly expressed, and MYC was lowly expressed in SW480 compared with NCM460 (Supplementary Figure S1A). Subsequently, we selected the PDK4 with the greatest difference in expression for functional validation, using siRNA to transfuse SW480 cells and downregulate PDK4 expression. siRNA NC was transfected into SW480 cells (Supplementary Figures S1B, C). CCK-8 demonstrated that downregulation of PDK4 hindered proliferation of SW480 cells (Supplementary Figure S1D). Transwell assay revealed that downregulation of PDK4 inhibited the migration and invasion of SW480 cells (Supplementary Figure S1E).

The correlation analysis of 55 ARGs revealed that there were significant positive correlations among the expression levels of CCL2, DLC1, CXCL12, CTSL, DCN, KRT18, NUPR1, and ITGA6, and a negative correlation among the expression levels of TBC1D10C, CDKN1A, MYC, and SMYD3 (Figure 5C). In addition, we performed KEGG pathway enrichment analysis for the 55 ARGs and validated that there were several pathways associated with metabolic pathways, such as proteoglycans in cancer, lipid and atherosclerosis, human cytomegalovirus infection, fluid shear stress, and atherosclerosis. (Figure 5D, Supplementary Table S3).

### 3.4 Construction and evaluation of a prognostic model for CRC patients

**FIGURE 6:**
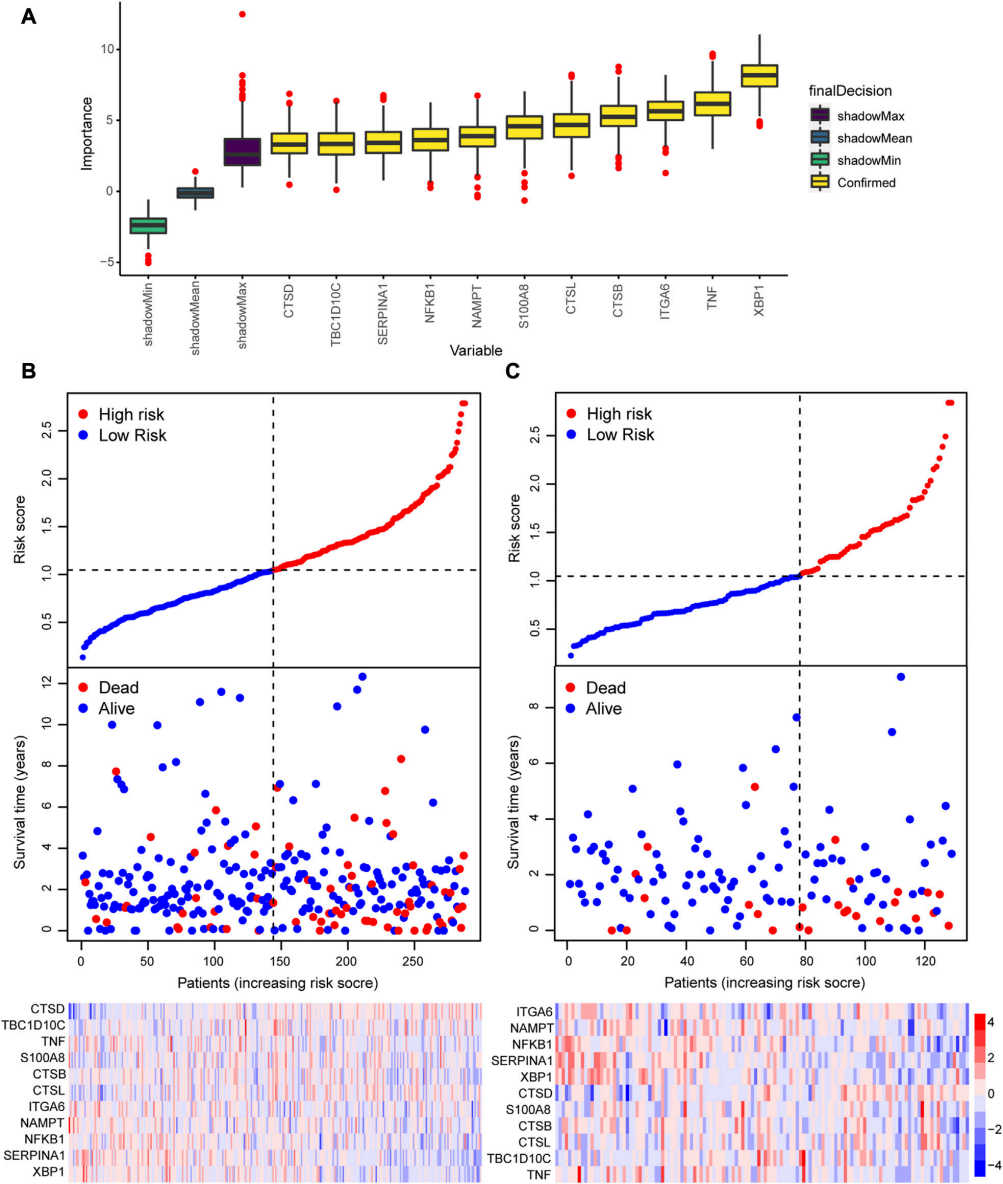
Construction of a prognostic model based on the 55 autophagy-related genes (ARGs). **(A)** Screening of the 11 hub ARGs associated with prognosis based on the Boruta feature. Risk score, survival status, and expression heatmap in the **(B)** training cohort and **(C)** validating cohort.

We performed a survival analysis for the 417 patients with survival information obtained from TCGA and screened 27 ARGs that are significantly related to the OS of the patients with CRC (Supplementary Table S4). Next, we identified 11 important ARGs based on the Boruta feature, which were CTSB, CTSD, ITGA6, NAMPT, NFKB1, SERRINA1, CTSL, S100A8, TBC1D10C, TNF and XBP1 (Figure 6A), and then calculated the regression coefficients of these 11 ARGs according to the multivariate Cox regression model (Table 1). Finally, we calculated the risk score for each patient based on the regression coefficients and expression levels of the 11 ARGs. The 417 patients were divided into high- and low-risk groups according to the median risk score (training cohort: 144 patients in the high-risk group, 144 patients in the low-risk group; validation cohort: 51 patients in the high-risk group, 78 patients in the low-risk group). The risk score distributions and survival states of the patients in the high-risk and low-risks group are shown in Figures 6B, C, respectively, the mortality risk increased and the survival time decreased with the increase in the risk score.

**TABLE 1:** Multivariate Cox regression analysis of 11 hug autophagy-related genes.

Gene	Coefficient
CTSB	−0.503
CTSD	0.463
ITGA6	0.022
NAMPT	−0.00183
NFKB1	−0.21
SERPINA1	−0.149
CTSL	0.168
S100A8	0.173
TBC1D10C	0.039
TNF	−0.434
XBP1	−0.039

**FIGURE 7:**
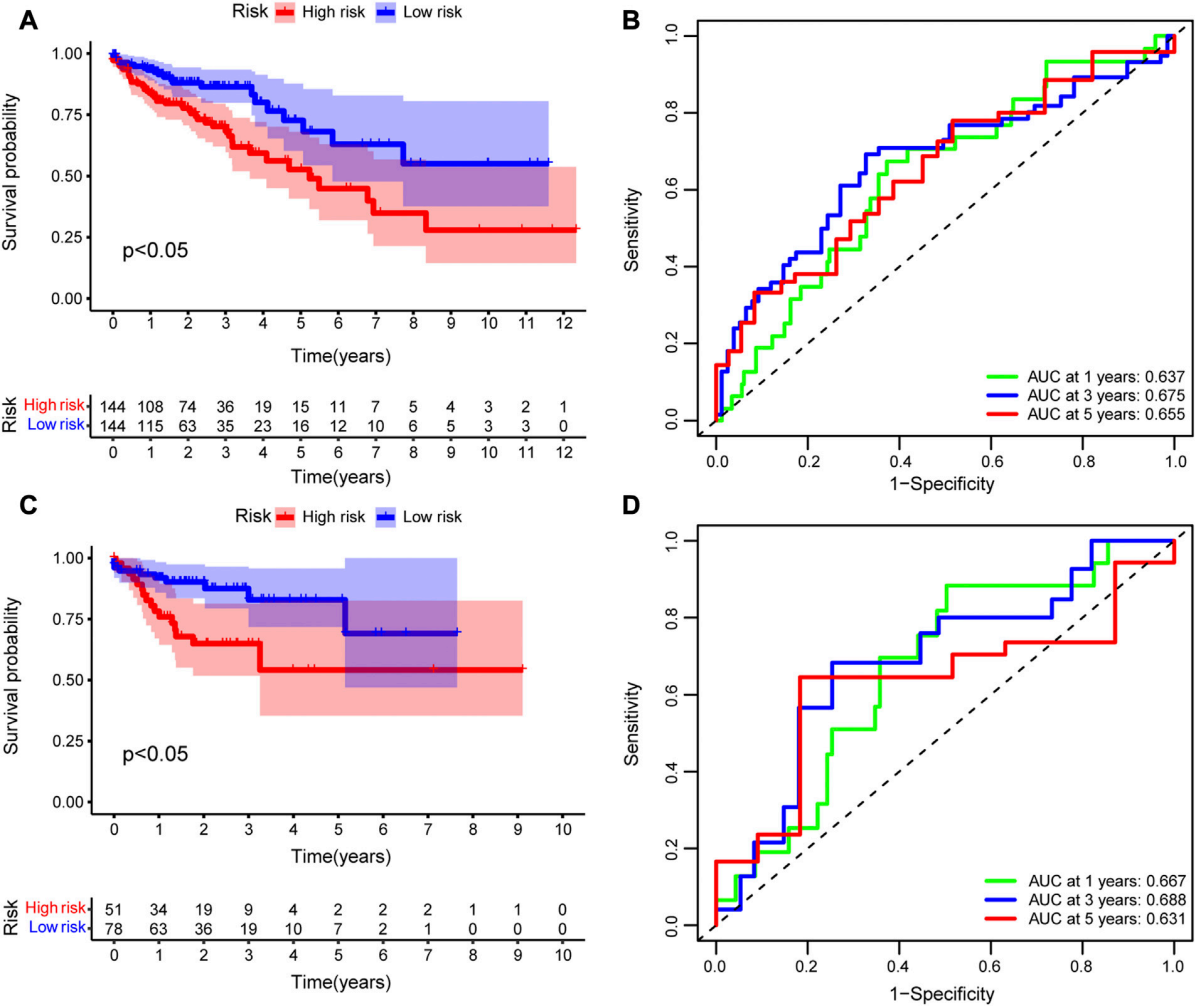
Evaluation of the prognostic model. Kaplan–Meier (K–M) survival analysis between high-risk and low-risk groups in the **(A)** training cohort and **(C)** validating cohort. Time-dependent receiver operating characteristic (ROC) analysis of 1-, 3-, and 5-year overall survival rates based on risk score of patients with CRC in the **(B)** training cohort and **(D)** validating cohort.

**FIGURE 8:**
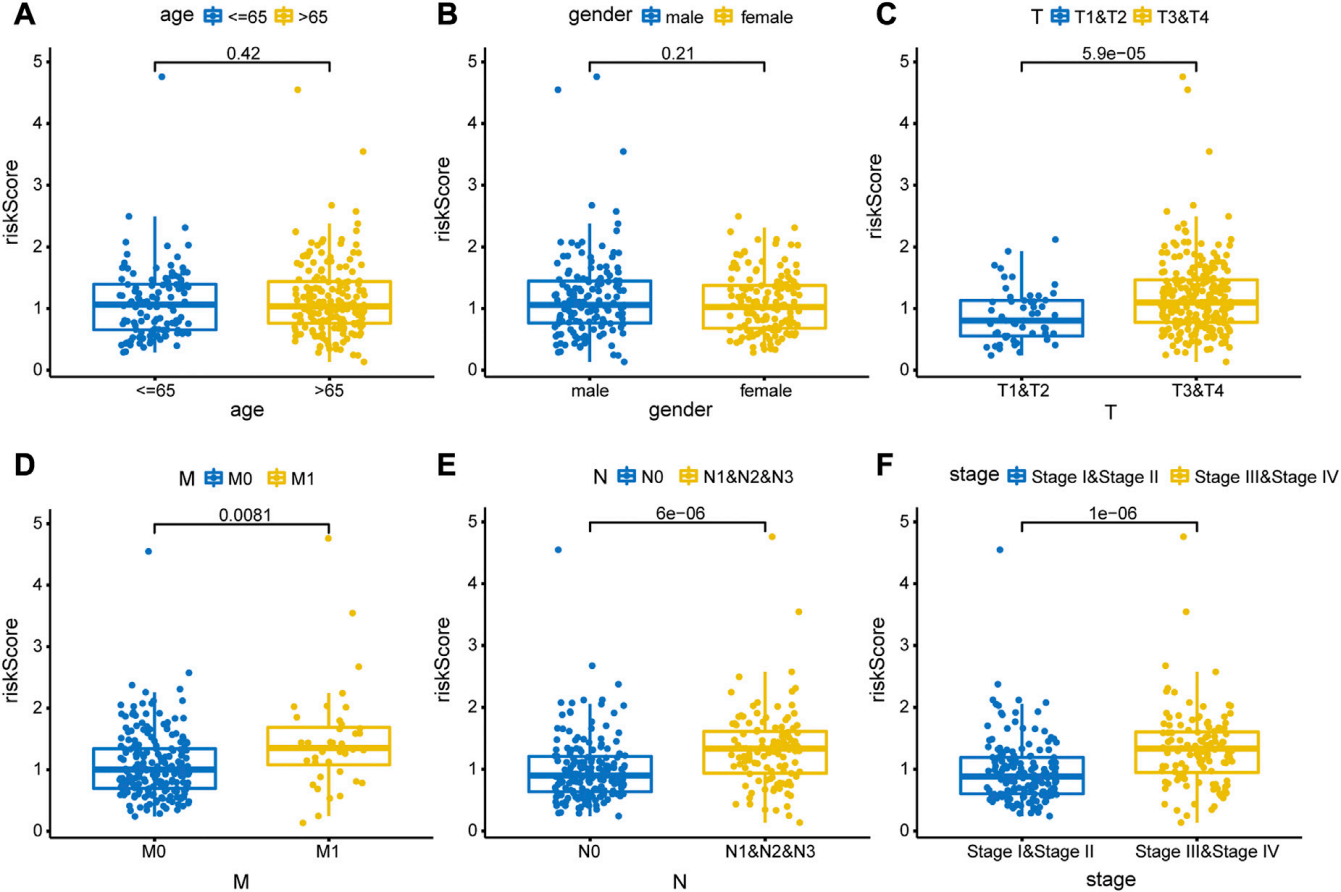
Correlations between risk score and clinical feature. The clinical features included **(A)** age (>65 years vs. < 65 years old), **(B)** sex (male vs. female), **(C)** T stage (T1&T2 vs. T3&T4), **(D)** M stage (M0 vs. M1), **(E)** N stage (N0 vs*.* N1&N2), and **(F)** pathological stage (stage Ⅰ& stage Ⅱ vs. stage Ⅲ & stage Ⅳ).

To evaluate the accuracy of the prognostic model, we performed survival analysis for patients in the training cohort and validation cohorts. We found that patients in the high-risk group had a poor prognosis, and there were significant differences in OS between the two groups (*p* < 0.05, Figures 7A, C). Time-depended ROC analysis revealed that the area under the curve (AUC) of 1-, 3-, and 5-year survival rates was more than 0.6 (Figures 7B, D), indicating that the risk score was a risk factor for patients with CRC. The risk score was not significantly affected by age and sex (Figures 8A, B), but was affected by the pathological stage. As shown in Figures 8C–F, the patients in the advanced stage had significantly higher risk scores (*p* < 0.05).

### 3.5 Immune infiltration associated with different risk groups and ARGs expression levels

**FIGURE 9:**
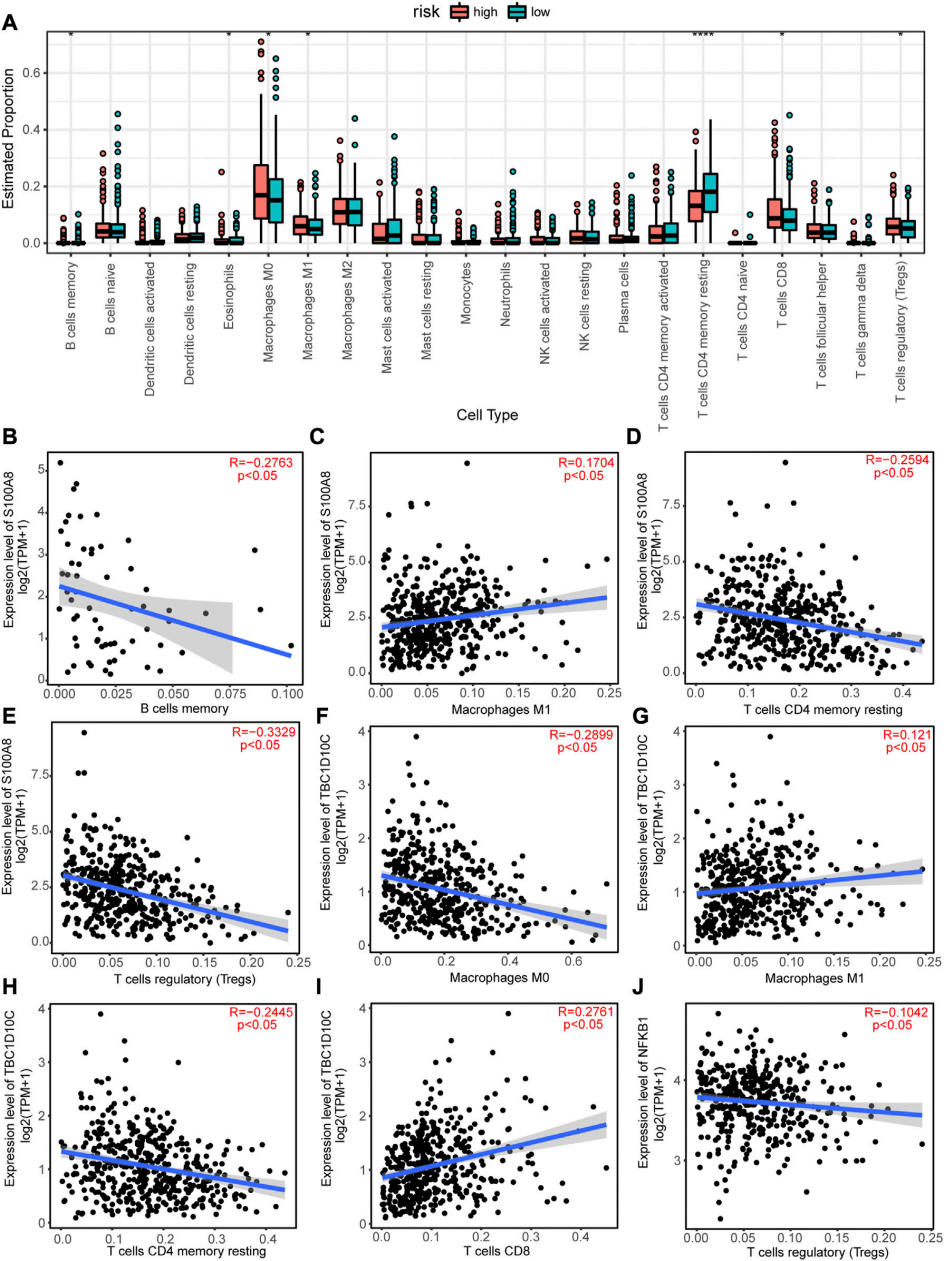
Immune infiltration analysis of the high-risk and low-risk groups and correlations between autophagy-related genes (ARGs) expression levels and immune cell infiltrations. **(A)** Differences in immune cell infiltrations between the high-risk and low-risk groups. *p < 0.05; **p < 0.01; ***p < 0.001; ****p < 0.0001. **(C)** S100A8 expression level positively correlated with macrophage M1 cell infiltration, but negatively correlated with **(B)** B cells memory cell infiltration, **(D)** T cells CD4 memory resting cell infiltration, and **(E)** T cells regulatory (Tregs) cell infiltration. TBC1D10C expression level is positively correlated with **(G)** Macrophages M1 and **(I)** T cells CD8 cell infiltration8, but negatively correlated with **(F)** Macrophages M0 and **(H)** T cells CD4 memory resting cell infiltration. **(J)** NFKB1 expression level negatively correlated with Treg cell infiltration.

As patients in the high-risk group had a poorer prognosis than those in the low-risk group, we speculated that there would be a significant difference in immune cell infiltration between the two groups. Therefore, we calculated the enriched proportion of different immune cells in the CRC tissue samples from TCGA using the CIBERSORT algorithm. As shown in Figure 9A, there were significant differences in enrichment of memory B cells, eosinophils, M0 macrophages, M1 macrophages, resting memory CD4^+^ T cells, CD8^+^ T cells, and regulatory T cells (Tregs) between the high- and low-risk group (*p* < 0.05).

In addition, the expression levels of the11 hub ARGs significantly correlated with immune cell infiltration in CRC tissues. For example, the expression level of S100A8 showed a significant negative correlation with the enrichment of memory B cells (Figure 9B), resting memory CD4^+^ T cells (Figure 9D), and Tregs (Figure 9E), but a positive correlation with the enrichment of M1 macrophages (Figure 9C). The expression level of TBC1D10C was significantly positively correlated with the enrichment of M1 macrophages (Figure 9G) and CD8^+^ T cells (Figure 9I), but significantly negatively correlated with the enrichment of M0 macrophages (Figure 9F) and resting memory CD4^+^ T cells (Figure 9H). The expression level of NFKB1 was significantly negatively correlated with the enrichment of Tregs (Figure 9J). Similarly, the expression levels of CTSB, CTSD, ITGA6, NAMPT, SERPINA1, TNF, XBP1, and CTSL was significantly correlated with the enrichments of various immune cells (Supplementary Figures S2-S3).

### 3.6 Prediction of drug sensitivity

**FIGURE 10:**
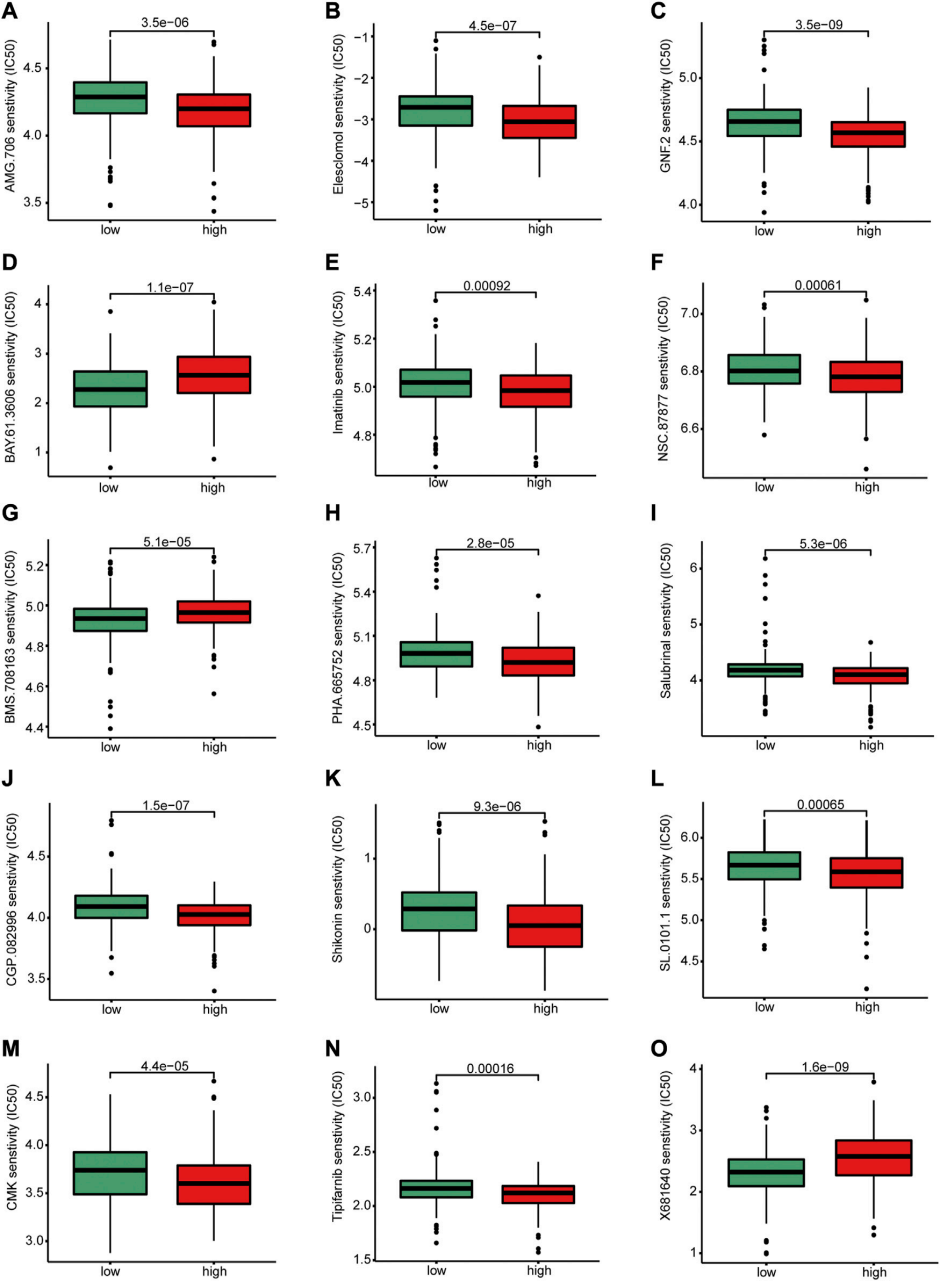
Differences in drug sensitivity between high and low-risk groups. There were significant differences between high-risk and low-risk group in the sensitivity of **(A)** AMG.706, **(B)** elesclomol, **(C)** GNF.2, **(D)** BAY.61.3606, **(E)** Imatinib, **(F)** NSC.8787, **(H)** PHA.665752, **(I)** salubrinal, **(J)** CGP.082996 **(K)** Shikonin, **(L)** SL.0101.1, **(M)** CMK, **(N)** Tipifarnib and **(O)** X681640.

To investigate whether the groups had different response to chemotherapies, we performed a drug sensitivity analysis between the two risk groups. Statistical differences in drug sensitivity between the high- and low-risk groups were noted (Figures 10A–O). For example, the IC_50_ values of AMG.706, elesclomol, GNF.2, imatinib, NSC.87877, PHA.665752, salubrinal, CGP.082996, shikonin, SL.0101.1, CMK, and tipifarnib were significantly lower in the high-risk group than in the low-risk group, whereas the IC_50_ values of BAY.61.3606 and BMS.708163 were significantly higher in the high-risk group than in the low-risk group.

## 4 Discussion

CRC has become an important public health issue owing to its high morbidity and mortality rates in the recent decades. Previous studies have mainly focused on screening the biomarkers for CRC based on the differentially expressed genes between tumor and normal tissues (Vayrynen et al., 2021; Xiao et al., 2022). However, tumor tissues contain various cell populations, and the differentially expressed genes between tumor and normal tissues cannot reflect the differences among the cell populations. ScRNA-seq analysis can identify cell subpopulations and is suitable for investigating the heterogeneity of cell populations (Deng et al., 2022). Moreover, scRNA-seq can help to identify hub genes associated with the tumor initiation and development of cancer, which might have potential value in precision therapy for patients with CRC (Mei et al., 2021). In our study, we analyzed the scRNA-seq data based on the GEO dataset GSE132257 and obtained 16,270 cells with superior quality to investigate the different genomic features between CRC and normal tissues at the single-cell level. Based on the DEGs among single cells and tissue samples, integrated with clinical information, we identified the 11 pivotal ARGs. Next, we constructed and validated a prognostic model based on the risk score associated with Cox regression coefficients and expression levels of the ARGs; the risk score of the patients with advanced tumors was significantly higher. We divided the patients into high- and low-risk groups, and found that there were significant differences in immune cell infiltration and drug sensitivities between the two groups. In addition, our findings revealed that the scRNA-seq method combined with large cohort population validation is an effective method to understand the genetic features with potential clinical value in personalized treatments for patients with CRC.

In the present study, we demonstrated that the cells in the CRC tissues presented significant heterogeneity. We identified, using PCA linear dimensionality reduction and tSNE, 15 cell clusters in CRC tissues and annotated them as T cells, B cells, epithelial cells, myeloid cells, fibroblasts, mast cells, and endothelial cells. The proportion of the different cell types in tumor tissues is altered during the development of the tumor, especially in the tumor microenvironment (TME). In CRC, immune cell infiltration in the TME is decreased and reflects a poor prognosis for patients with CRC (Khaliq et al., 2022). Consistent with these theories of tumor development, our trajectories reconstruction and analysis revealed that the proportions of immune cells decreased with the stages of cell differentiation and maturation.

In addition, KEGG pathway enrichment analysis of the DEGs revealed that many differentially enriched pathways in the seven cell types, most of which were associate with cell metabolism and immune responses, such as primary immunodeficiency, N-glycan biosynthesis, linoleic acid metabolism, and arachidonic acid metabolism. Similarly, using c7.immunesigdb.v7.5.1.symbols.gmt as a reference gene set, we identified many signaling pathways involved in the cellular immune response. Moreover, many studies have shown that autophagy is closely related to tumor formation and development. Autophagy is attenuated in various tumors, and increased autophagy activity can effectively suppress tumor formation in animal models. However, autophagy can also promote tumor procession and metastasis following tumor development (Thorburn et al., 2014; Yuan et al., 2019). In addition, the interleukin (IL)-17 signaling pathway plays a tumorigenic role in CRC and is associated with the activation of nuclear factor κ-light-chain-enhancer of activated B (NF-κB) cells and mitogen-activated protein kinases (MAPKs), which upregulate the survival pathways required for the growth and development of tumor cells (Wang et al., 2014). Consistent with these findings, in our study, the KEGG pathway enrichment analysis demonstrated that the differentially expressed ARGs were enriched in various pathways associated with tumor initiation and development, such as proteoglycans in cancer, IL-17 signaling pathway, and apoptosis.

The prognosis of patients with CRC is roughly predicted based on their general profile, pathological type, and pathological stage. However, owing to the development of RNA-seq technology, the prognosis of patients with CRC can be more accurately predicted by integrating gene expression features and clinical information. Therefore, in the present study, we demonstrated the potential prognostic value of ARGs based on RNA-seq and clinical data from TCGA and constructed a prognostic model, which exhibited excellent performance in predicting the prognosis of patients with CRC. We identified 11 hub ARGs that have been previously shown to be closely related to the carcinogenesis of CRC. For example, CTSD, which is highly expressed in CRC tissues, is closely associated with a poor prognosis by promoting β-catenin pathway (Ding et al., 2022). Moreover, NAMPT is overexpressed in CRC tissues and is associated with a poor prognosis by stabilizing the protein components of Smad2, Smad3, and Smad4 in the TGF-β signaling pathway, thereby increasing the activity of the TGF-β signaling (Lv et al., 2021). S100A8 promotes CRC metastasis by increasing epithelial-mesenchymal transition and promoting the TGF-β signaling pathway (Li et al., 2021). Therefore, these reports indicate that the 11 hub ARGs identified in this study might help reveal the molecular mechanism associated with the initiation and development of CRC.

Autophagy acts as a double-edge sword; it inhibits the tumor procession at the early stages, but promotes tumor formation and development during advanced stages. Numerous studies have shown that when autophagy is involved in tumor procession, it is associated with immune cell infiltration in the TME. Chloroquine (CQ) is an autophagy inhibitor that blocks autophagosome-lysosome fusion. In Melanoma, CQ decreases the infiltration of lymphocytes and macrophages in the TME and enhances the anticancer effects of the MEK inhibitor trametinib (TRA) (Degan et al., 2022; Li et al., 2022). In present study, we calculated the risk score of patients with CRC based on the expression levels of ARGs and coefficients of Cox regression survival analysis and divided the patients into high- and low-risk groups according to the median of risk score. We found significant differences in immune cell infiltration between the high- and low-risk groups. M0 macrophages, M1 macrophages, CD8^+^ T cells, and Tregs were significantly more enriched in the high-risk group than in low-risk group; whereas B cells memory, eosinophils, and resting CD4^+^ T cells were significantly less enriched in high-risk group than in low-risk group. Tregs play a role in immunosuppression in the TME (Kugel et al., 2018), which might be associated with the poor prognosis of patients with CRC in the high-risk group. Zhang et al. (2019) reported that memory B cells played a role in tumor-killing in hepatocellular carcinoma (HCC), and high infiltration of memory B cells in HCC indicates a good prognosis. Therefore, our study is consistent with previous studies, memory B cells, eosinophils, and resting CD4^+^ T cells are related to the antitumoral immune response in the TME, which might be associated with a good prognosis of patients with CRC in the low-risk group. Moreover, our study revealed that the expression levels of the 11 hub ARGs were closely related to immune cell infiltration; S100A8 and TBC1D10C expression levels were positively correlated with the macrophage M1 immune filtrating level in CRC tissues whereas NFKB1 expression levels were negatively correlated with the level of infiltrating Tregs. Collectively, these results indicate that the 11 identified hub ARGs are associated with altered immune cell infiltration in CRC tissues and have potential value in predicting the prognosis of patient with CRC.

Drug sensitivity prediction can guide the clinical decision-making in selecting chemotherapy for different patient clusters. Therefore, we predicted the response to drugs of patients with CRC in the high- and low-risk groups and showed that there were significant differences in drug sensitivity between the two groups. Patients in the high-risk group were sensitive to several kinds of anticancer drugs, including elesclomol which is an oxidative stress inducer that mediates cell apoptosis (Buccarelli et al., 2021); and imatinib which is a tyrosine kinase inhibitor that is widely used in the treatment of chronic myeloid leukemia (Cortes et al., 2018). S100A8 has been demonstrated inducing apoptosis of imatinib-resistant human eosinophilic leukemia cell lines (Lee J. S. et al., 2020). Moreover, shikonin promoted CRC cells apoptosis by endoplasmic reticulum stress (Qi et al., 2022). In our study, the IC_50_ value of shikonin was significantly lower in the high-risk group than in the low-risk group, which indicating shikonin had a good therapeutic effect on patients in the high-risk group. Therefore, our drug sensitivity analysis provides a potential predictive tool for anticancer drug selection and is useful for individualized therapy.

Despite these promising results, this study has some limitations. First, we studied the prognostic performance of the genes only at the RNA-level; the protein level requires further investigation. Second, the candidate genes in this study were restricted to ARGs; however, there are several interactions among different types of molecules. Finally, the analysis was based on bioinformatic methods; supplemental cell or animal experiments are needed to reveal the potential role of ARGs in the progression of CRC.

In conclusion, our scRNA-seq integrated with validated cohorts revealed that the 11 hub ARGs had powerful performance in predicting prognosis, immune response and drug sensitivity in patients with CRC. This 11-hub ARG model might service as a prognostic model and is useful for clinical decision-making to select appropriate patients who might benefit from anticancer drug therapy.

## Data Availability

The original contributions presented in the study are included in the article/Supplementary Material, further inquiries can be directed to the corresponding author.
