# Supplementary material for: Prognostic value of autophagy-related genes based on single-cell RNA-sequencing in colorectal cancer
Source: Front Genet. 2023 Mar 30;14:1109683. doi: 10.3389/fgene.2023.1109683 (PMC10097963; doi:10.3389/fgene.2023.1109683)
Supplement: Supplementary file 1 [file Table1.docx]

**Supplementary figure legends**

**Figure S1.** PDK4 regulates colon cells proliferation, migration, and invasion. **(A)** qRT-PCR validation for 8 ARGs. qRT-PCR **(B)** and western blot **(C)** validation for the transfection efficiency of PDK4 si-RNA in colon cells. **(D)** CCK-8 assay was used to determine cell growth. **(E)** Transwell assays was used to determine cell migration and invasion. Scale bar=20x. * p <0.05; **p < 0.01; n=3.

**Fig. S2** Correlations between CTSB, CTSD, CTSL, and ITGA6 expression levels and immune cell infiltrations.

**Fig. S3** Correlations between ITGA6, NAMPT, NFKB1, SERPINA1, TBC1D10C, TNF, and XBP1 expression levels and immune cell infiltrations.

**Supplementary table legends**

**Table S1.** Specific information on primer sequences

**Table S2** Differential expression of ARGs between CRC tissues and normal adjacent tissues based on RNA-seq from TCGA

**Table S3** KEGG pathway enrichment analysis

**Table S4** Survival analysis of the 55 ARGs
