# Supplementary material for: Prognostic value of autophagy-related genes based on single-cell RNA-sequencing in colorectal cancer
Source: Front Genet. 2023 Mar 30;14:1109683. doi: 10.3389/fgene.2023.1109683 (PMC10097963; doi:10.3389/fgene.2023.1109683)
Supplement: Supplementary file 5 [file Table2.DOCX]

| Table S1. Specific information on primer sequences | |
| --- | --- |
| **Genes** | **Primer sequence (5'to3')** |
| PDK4-F | GGAAGCATTGATCCTAACTGTGA |
| PDK4-R | GGTGAGAAGGAACATACACGATG |
| CCDN1-F | CAATGACCCCGCACGATTTC |
| CCDN1-R | CATGGAGGGCGGATTGGAA |
| SMYD3-F | CCCCACCTCTTACTGCGAG |
| SMYD3-R | TGGCAACGGAAACAGTCACAT |
| CXCL12-F | TGCCCTTCAGATTGTAGCCC |
| CXCL12-R | GCGTCTGACCCTCTCACATC |
| Homo-MYC-F | GGAACTTACAACACCCGAGC |
| Homo-MYC-R | GTGAAGCTAACGTTGAGGGG |
| Homo-DCN-F | GCCCATGAGAATGAGATCACC |
| Homo-DCN-R | AATGCGGATGTAGGAGAGCT |
| BID-F | CTTGCTCCGTGATGTCTTTCA |
| BID-R | TCCGTTCAGTCCATCCCATT |
| TNFSF10-F | TGAGAACCTCTGAGGAAACCA |
| TNFSF10-R | TTTATTTTGCGGCCCAGAGC |
| GAPDH-F | ACAACTTTGGTATCGTGGAAGG |
| GAPDH-R | GCCATCACGCCACAGTTTC |
