# Supplementary material for: Prognostic value of autophagy-related genes based on single-cell RNA-sequencing in colorectal cancer
Source: Front Genet. 2023 Mar 30;14:1109683. doi: 10.3389/fgene.2023.1109683 (PMC10097963; doi:10.3389/fgene.2023.1109683)
Supplement: Supplementary file 6 [file Table5.DOCX]

**Table S4 Survival analysis of the 55 ARGs**

| gene_ids | P value |
| --- | --- |
| BAG3 | 0.020525096 |
| BID | 0.085885388 |
| CCL2 | 0.075621089 |
| CDKN1A | 0.067745969 |
| CTSB | 0.004193747 |
| CTSD | 0.001138651 |
| CXCR4 | 0.231121162 |
| DLC1 | 0.05764827 |
| FKBP1A | 0.029959498 |
| FOS | 0.064606184 |
| GABARAPL2 | 0.119472424 |
| HIF1A | 0.166284909 |
| HSPA8 | 0.001382817 |
| IFNG | 0.2326162 |
| ITGA6 | 0.002256653 |
| ITGB1 | 0.165656424 |
| MYC | 0.038520252 |
| NAMPT | 0.005155151 |
| NFE2L2 | 0.173521399 |
| NFKB1 | 0.013256602 |
| PPP1R15A | 0.136776259 |
| RAB11A | 0.075025651 |
| RAC1 | 0.454494718 |
| SERPINA1 | 0.00418049 |
| TNFSF10 | 0.084782011 |
| ABL2 | 0.036297651 |
| APP | 0.117266202 |
| BST2 | 0.011877848 |
| CCND1 | 0.017350328 |
| CTNNB1 | 0.019353556 |
| CTSL | 0.001481739 |
| CXCL12 | 0.041410637 |
| CYB5A | 8.07E-05 |
| DCN | 0.201113988 |
| ENG | 0.026557759 |
| FN1 | 0.002059373 |
| HSPG2 | 0.101028783 |
| IL6 | 0.08399602 |
| KDR | 0.068564975 |
| KRT18 | 0.031285728 |
| MITF | 0.240739442 |
| NDRG1 | 0.02832091 |
| NUPR1 | 0.167512039 |
| PDK4 | 0.016372537 |
| PSAP | 0.185695614 |
| PTPN22 | 0.103799219 |
| QSOX1 | 0.069237851 |
| RASIP1 | 0.107993142 |
| S100A8 | 0.036250406 |
| S100A9 | 0.371792026 |
| SMYD3 | 0.013016278 |
| SOD2 | 0.187779157 |
| TBC1D10C | 0.005439851 |
| TNF | 0.001421679 |
| XBP1 | 0.000427194 |
