# Supplementary material for: Prognostic value of autophagy-related genes based on single-cell RNA-sequencing in colorectal cancer
Source: Front Genet. 2023 Mar 30;14:1109683. doi: 10.3389/fgene.2023.1109683 (PMC10097963; doi:10.3389/fgene.2023.1109683)
Supplement: Supplementary file 7 [file Table3.DOCX]

**Table S2 Differential expression of ARGs between CRC tissues and normal adjacent tissues based on RNA-seq from TCGA**

|  | baseMean | log2FoldChange | lfcSE | stat | pvalue | padj | change |
| --- | --- | --- | --- | --- | --- | --- | --- |
| CCND1 | 6928.918029473 | 1.907438591 | 0.11188715 | 17.048018771 | 3.61583E-65 | 1.98871E-63 | UP |
| PDK4 | 1049.466755952 | -3.184444738 | 0.20245462 | -15.72552714 | 1.01106E-55 | 2.78041E-54 | DOWN |
| MYC | 8440.158173917 | 2.101068822 | 0.143838878 | 14.607604048 | 2.51198E-48 | 4.60529E-47 | UP |
| CXCL12 | 1052.490016594 | -2.510107 | 0.184851884 | -13.577307763 | 5.45966E-42 | 7.50703E-41 | DOWN |
| SMYD3 | 728.401620925 | 1.23388717 | 0.109490763 | 11.269216108 | 1.8622E-29 | 2.04842E-28 | UP |
| TNFSF10 | 2576.507455937 | -1.271118099 | 0.114183234 | -11.132172912 | 8.74767E-29 | 8.0187E-28 | DOWN |
| BID | 2042.786982062 | 1.169758333 | 0.114694774 | 10.198911102 | 2.00508E-24 | 1.57542E-23 | UP |
| DCN | 7046.589896509 | -1.732717591 | 0.192842951 | -8.984175761 | 2.60681E-19 | 1.79218E-18 | DOWN |
| CTNNB1 | 21005.462342872 | 0.7471671 | 0.087706976 | 8.518916619 | 1.61053E-17 | 9.84211E-17 | NOT |
| ITGA6 | 14462.530771651 | 1.003001244 | 0.121181172 | 8.27694661 | 1.26374E-16 | 6.95056E-16 | UP |
| KRT18 | 43426.036095352 | 1.162685715 | 0.149834003 | 7.760024901 | 8.49127E-15 | 4.24564E-14 | UP |
| HSPA8 | 52301.921488948 | 0.696054037 | 0.097216095 | 7.15988379 | 8.07455E-13 | 3.70083E-12 | NOT |
| NDRG1 | 14013.444567386 | -0.972854635 | 0.152838521 | -6.36509743 | 1.95166E-10 | 8.25702E-10 | NOT |
| CDKN1A | 5986.617703446 | -0.917878483 | 0.155100337 | -5.917832863 | 3.26211E-09 | 1.28154E-08 | NOT |
| ABL2 | 994.349368595 | 0.831498642 | 0.141218504 | 5.888076542 | 3.90716E-09 | 1.43263E-08 | NOT |
| CTSB | 30931.615122324 | 0.4475866 | 0.093283675 | 4.798130865 | 1.60153E-06 | 5.50526E-06 | NOT |
| MITF | 252.328160825 | -0.965482823 | 0.201917159 | -4.781298811 | 1.74166E-06 | 5.63479E-06 | NOT |
| DLC1 | 649.6394135 | -0.691711474 | 0.15173964 | -4.558486478 | 5.15236E-06 | 1.57433E-05 | NOT |
| BST2 | 2722.781747122 | 1.030555132 | 0.235759155 | 4.371846605 | 1.232E-05 | 3.56632E-05 | UP |
| NAMPT | 4635.886534998 | 0.654162674 | 0.152054261 | 4.302228842 | 1.69089E-05 | 4.64993E-05 | NOT |
| CYB5A | 2032.708594071 | -0.455915964 | 0.109115819 | -4.178267751 | 2.93738E-05 | 7.69313E-05 | NOT |
| IFNG | 12.926763836 | 1.137322978 | 0.312038604 | 3.643176855 | 0.000269294 | 0.000673234 | UP |
| S100A9 | 1094.725582216 | 0.776747439 | 0.229120618 | 3.390435187 | 0.000697818 | 0.001668694 | NOT |
| SERPINA1 | 12481.880496642 | 0.389142096 | 0.268469839 | 3.356811723 | 0.000788468 | 0.001806905 | NOT |
| CTSD | 11084.124571579 | -0.409721218 | 0.122629533 | -3.341116259 | 0.000834423 | 0.00183573 | NOT |
| CCL2 | 472.723146462 | -0.613114578 | 0.187930371 | -3.262358894 | 0.001104892 | 0.002337271 | NOT |
| HSPG2 | 8636.817164879 | -0.5491422 | 0.170401865 | -3.222564287 | 0.001270487 | 0.002588028 | NOT |
| TBC1D10C | 197.823639208 | -0.573580341 | 0.180715696 | -3.173886279 | 0.001504126 | 0.002954533 | NOT |
| PSAP | 38237.171656391 | -0.230730266 | 0.074442273 | -3.099451108 | 0.001938796 | 0.003677026 | NOT |
| XBP1 | 10018.860056871 | 0.349783262 | 0.113725751 | 3.075681261 | 0.002100222 | 0.003850408 | NOT |
| FOS | 10816.851616329 | -0.609348744 | 0.206967703 | -2.944024414 | 0.003239744 | 0.005747934 | NOT |
| RAC1 | 13539.162001489 | 0.241567061 | 0.090890321 | 2.657789306 | 0.007865505 | 0.013518837 | NOT |
| PPP1R15A | 3057.320775462 | 0.332301547 | 0.126182137 | 2.633515427 | 0.0084506 | 0.014084333 | NOT |
| IL6 | 123.390792343 | 0.727699137 | 0.285407063 | 2.550148136 | 0.010767716 | 0.017418363 | NOT |
| SOD2 | 12545.053909808 | 0.311624116 | 0.127487096 | 2.444370458 | 0.014510518 | 0.022802243 | NOT |
| GABARAPL2 | 2211.150579022 | -0.20630179 | 0.089160928 | -2.313813602 | 0.020677949 | 0.031591311 | NOT |
| CTSL | 2094.33921528 | -0.313147475 | 0.142431374 | -2.198576486 | 0.02790805 | 0.04148494 | NOT |
| APP | 37588.449743306 | 0.17661811 | 0.088930482 | 1.986026127 | 0.047030423 | 0.068070349 | NOT |
| PTPN22 | 152.486067077 | -0.349022974 | 0.179940982 | -1.939637845 | 0.052423718 | 0.073930885 | NOT |
| FN1 | 37428.416316566 | 0.459640237 | 0.25135396 | 1.828814967 | 0.067427332 | 0.092712582 | NOT |
| ITGB1 | 10255.838094064 | -0.169746887 | 0.098556379 | -1.722331314 | 0.085009518 | 0.114037159 | NOT |
| KDR | 583.577809519 | 0.171160858 | 0.126741962 | 1.350467897 | 0.176865944 | 0.231610165 | NOT |
| RASIP1 | 257.688732113 | -0.186050213 | 0.139533932 | -1.333369718 | 0.182410505 | 0.233315762 | NOT |
| NFE2L2 | 3811.044250964 | -0.111528243 | 0.091637215 | -1.217061976 | 0.223580637 | 0.273456364 | NOT |
| TNF | 38.705861403 | -0.305257016 | 0.168867227 | 1.216651012 | 0.223737025 | 0.273456364 | NOT |
| CXCR4 | 1245.09271644 | 0.194453721 | 0.177820436 | 1.093549541 | 0.27415259 | 0.326138198 | NOT |
| HIF1A | 3438.738415138 | 0.141167692 | 0.130319614 | 1.083244495 | 0.278699915 | 0.326138198 | NOT |
| NFKB1 | 2550.059032137 | -0.064937293 | 0.064290967 | -1.010052796 | 0.312469996 | 0.358038537 | NOT |
| ENG | 2216.890470829 | 0.08025423 | 0.123462635 | 0.650029648 | 0.515673071 | 0.578816712 | NOT |
| RAB11A | 6309.880149911 | -0.051743397 | 0.0846097 | -0.611551571 | 0.540834488 | 0.594917937 | NOT |
| BAG3 | 1821.624961897 | 0.060371211 | 0.104972297 | 0.575116406 | 0.565212573 | 0.609542971 | NOT |
| NUPR1 | 264.439098038 | 0.113073364 | 0.251230733 | 0.450089315 | 0.652646041 | 0.690298697 | NOT |
| FKBP1A | 6124.571210591 | -0.028107369 | 0.106728491 | -0.263353217 | 0.792278345 | 0.822175642 | NOT |
| S100A8 | 218.162163328 | -0.023503117 | 0.276873634 | -0.084885929 | 0.932352078 | 0.949617858 | NOT |
| QSOX1 | 10881.399213651 | -0.005905927 | 0.153736898 | -0.03841476 | 0.969356993 | 0.969356993 | NOT |
