# Supplementary material for: Prognostic value of autophagy-related genes based on single-cell RNA-sequencing in colorectal cancer
Source: Front Genet. 2023 Mar 30;14:1109683. doi: 10.3389/fgene.2023.1109683 (PMC10097963; doi:10.3389/fgene.2023.1109683)
Supplement: Supplementary file 8 [file Table4.DOCX]

**Table S3 KEGG pathway enrichment analysis**

| ID | Description | GeneRatio | BgRatio | P value | p.adjust | qvalue |
| --- | --- | --- | --- | --- | --- | --- |
| hsa05205 | Proteoglycans in cancer | 13/45 | 205/8146 | 3.99E-11 | 7.23E-09 | 2.65E-09 |
| hsa05417 | Lipid and atherosclerosis | 12/45 | 215/8146 | 1.12E-09 | 1.02E-07 | 3.73E-08 |
| hsa05163 | Human cytomegalovirus infection | 12/45 | 225/8146 | 1.89E-09 | 1.14E-07 | 4.18E-08 |
| hsa05418 | Fluid shear stress and atherosclerosis | 10/45 | 139/8146 | 2.90E-09 | 1.31E-07 | 4.81E-08 |
| hsa04657 | IL-17 signaling pathway | 8/45 | 94/8146 | 3.53E-08 | 1.28E-06 | 4.68E-07 |
| hsa05167 | Kaposi sarcoma-associated herpesvirus infection | 10/45 | 194/8146 | 7.19E-08 | 2.17E-06 | 7.95E-07 |
| hsa05222 | Small cell lung cancer | 7/45 | 92/8146 | 5.96E-07 | 1.20E-05 | 4.41E-06 |
| hsa04210 | Apoptosis | 8/45 | 136/8146 | 6.29E-07 | 1.20E-05 | 4.41E-06 |
| hsa05323 | Rheumatoid arthritis | 7/45 | 93/8146 | 6.41E-07 | 1.20E-05 | 4.41E-06 |
| hsa05135 | Yersinia infection | 8/45 | 137/8146 | 6.65E-07 | 1.20E-05 | 4.41E-06 |
| hsa04933 | AGE-RAGE signaling pathway in diabetic complications | 7/45 | 100/8146 | 1.05E-06 | 1.73E-05 | 6.35E-06 |
| hsa05160 | Hepatitis C | 8/45 | 157/8146 | 1.87E-06 | 2.83E-05 | 1.04E-05 |
| hsa05161 | Hepatitis B | 8/45 | 162/8146 | 2.37E-06 | 3.30E-05 | 1.21E-05 |
| hsa05164 | Influenza A | 8/45 | 171/8146 | 3.56E-06 | 4.60E-05 | 1.69E-05 |
| hsa05210 | Colorectal cancer | 6/45 | 86/8146 | 6.80E-06 | 8.21E-05 | 3.01E-05 |
| hsa05169 | Epstein-Barr virus infection | 8/45 | 202/8146 | 1.22E-05 | 0.000137984 | 5.06E-05 |
| hsa04061 | Viral protein interaction with cytokine and cytokine receptor | 6/45 | 100/8146 | 1.63E-05 | 0.000173278 | 6.35E-05 |
| hsa04151 | PI3K-Akt signaling pathway | 10/45 | 354/8146 | 1.73E-05 | 0.000173694 | 6.36E-05 |
| hsa05142 | Chagas disease | 6/45 | 102/8146 | 1.82E-05 | 0.000173694 | 6.36E-05 |
| hsa04932 | Non-alcoholic fatty liver disease | 7/45 | 155/8146 | 1.95E-05 | 0.000176501 | 6.47E-05 |
| hsa05145 | Toxoplasmosis | 6/45 | 112/8146 | 3.11E-05 | 0.000267936 | 9.82E-05 |
| hsa05216 | Thyroid cancer | 4/45 | 37/8146 | 4.70E-05 | 0.000386574 | 0.000141635 |
| hsa05132 | Salmonella infection | 8/45 | 249/8146 | 5.51E-05 | 0.000418985 | 0.00015351 |
| hsa05133 | Pertussis | 5/45 | 76/8146 | 5.65E-05 | 0.000418985 | 0.00015351 |
| hsa04621 | NOD-like receptor signaling pathway | 7/45 | 184/8146 | 5.88E-05 | 0.000418985 | 0.00015351 |
| hsa05140 | Leishmaniasis | 5/45 | 77/8146 | 6.02E-05 | 0.000418985 | 0.00015351 |
| hsa04612 | Antigen processing and presentation | 5/45 | 78/8146 | 6.40E-05 | 0.000426479 | 0.000156256 |
| hsa04380 | Osteoclast differentiation | 6/45 | 128/8146 | 6.60E-05 | 0.000426479 | 0.000156256 |
| hsa04068 | FoxO signaling pathway | 6/45 | 131/8146 | 7.51E-05 | 0.000468656 | 0.000171709 |
| hsa05130 | Pathogenic Escherichia coli infection | 7/45 | 197/8146 | 9.05E-05 | 0.000546146 | 0.0002001 |
| hsa04510 | Focal adhesion | 7/45 | 201/8146 | 0.000102716 | 0.000590445 | 0.000216331 |
| hsa05162 | Measles | 6/45 | 139/8146 | 0.000104388 | 0.000590445 | 0.000216331 |
| hsa05170 | Human immunodeficiency virus 1 infection | 7/45 | 212/8146 | 0.000143325 | 0.000786113 | 0.000288021 |
| hsa05144 | Malaria | 4/45 | 50/8146 | 0.00015549 | 0.000827757 | 0.000303278 |
| hsa05166 | Human T-cell leukemia virus 1 infection | 7/45 | 222/8146 | 0.000190766 | 0.000986135 | 0.000361306 |
| hsa05208 | Chemical carcinogenesis - reactive oxygen species | 7/45 | 223/8146 | 0.000196137 | 0.000986135 | 0.000361306 |
| hsa05146 | Amoebiasis | 5/45 | 102/8146 | 0.000229022 | 0.001120353 | 0.000410481 |
| hsa04620 | Toll-like receptor signaling pathway | 5/45 | 104/8146 | 0.000250801 | 0.001194607 | 0.000437687 |
| hsa05134 | Legionellosis | 4/45 | 57/8146 | 0.000259292 | 0.001203381 | 0.000440901 |
| hsa05213 | Endometrial cancer | 4/45 | 58/8146 | 0.000277381 | 0.00125515 | 0.000459869 |
| hsa04659 | Th17 cell differentiation | 5/45 | 108/8146 | 0.000299062 | 0.001320247 | 0.00048372 |
| hsa04066 | HIF-1 signaling pathway | 5/45 | 109/8146 | 0.000312159 | 0.001331212 | 0.000487737 |
| hsa05416 | Viral myocarditis | 4/45 | 60/8146 | 0.000316255 | 0.001331212 | 0.000487737 |
| hsa04668 | TNF signaling pathway | 5/45 | 112/8146 | 0.000354075 | 0.001456537 | 0.000533654 |
| hsa04670 | Leukocyte transendothelial migration | 5/45 | 114/8146 | 0.000384302 | 0.001545748 | 0.00056634 |
| hsa05165 | Human papillomavirus infection | 8/45 | 331/8146 | 0.000394449 | 0.001552069 | 0.000568656 |
| hsa05152 | Tuberculosis | 6/45 | 180/8146 | 0.000426282 | 0.001622801 | 0.000594571 |
| hsa05321 | Inflammatory bowel disease | 4/45 | 65/8146 | 0.000430356 | 0.001622801 | 0.000594571 |
| hsa04071 | Sphingolipid signaling pathway | 5/45 | 119/8146 | 0.000468422 | 0.001730293 | 0.000633954 |
| hsa01523 | Antifolate resistance | 3/45 | 30/8146 | 0.000576282 | 0.002086141 | 0.000764332 |
| hsa05202 | Transcriptional misregulation in cancer | 6/45 | 193/8146 | 0.000617456 | 0.002191363 | 0.000802884 |
| hsa04650 | Natural killer cell mediated cytotoxicity | 5/45 | 131/8146 | 0.000726701 | 0.002529478 | 0.000926764 |
| hsa05212 | Pancreatic cancer | 4/45 | 76/8146 | 0.000780264 | 0.002615329 | 0.000958219 |
| hsa05220 | Chronic myeloid leukemia | 4/45 | 76/8146 | 0.000780264 | 0.002615329 | 0.000958219 |
| hsa05100 | Bacterial invasion of epithelial cells | 4/45 | 77/8146 | 0.00081972 | 0.002697625 | 0.000988371 |
| hsa04140 | Autophagy - animal | 5/45 | 141/8146 | 0.001013284 | 0.003238421 | 0.001186511 |
| hsa04010 | MAPK signaling pathway | 7/45 | 294/8146 | 0.001036469 | 0.003238421 | 0.001186511 |
| hsa04936 | Alcoholic liver disease | 5/45 | 142/8146 | 0.001045978 | 0.003238421 | 0.001186511 |
| hsa04060 | Cytokine-cytokine receptor interaction | 7/45 | 295/8146 | 0.001057282 | 0.003238421 | 0.001186511 |
| hsa05143 | African trypanosomiasis | 3/45 | 37/8146 | 0.00107351 | 0.003238421 | 0.001186511 |
| hsa04810 | Regulation of actin cytoskeleton | 6/45 | 218/8146 | 0.00116724 | 0.003463451 | 0.001268958 |
| hsa05224 | Breast cancer | 5/45 | 147/8146 | 0.001221374 | 0.003565623 | 0.001306393 |
| hsa04512 | ECM-receptor interaction | 4/45 | 88/8146 | 0.001351501 | 0.003882884 | 0.001422633 |
| hsa05235 | PD-L1 expression and PD-1 checkpoint pathway in cancer | 4/45 | 89/8146 | 0.001409437 | 0.003986063 | 0.001460436 |
| hsa05219 | Bladder cancer | 3/45 | 41/8146 | 0.00145027 | 0.004028862 | 0.001476117 |
| hsa05410 | Hypertrophic cardiomyopathy | 4/45 | 90/8146 | 0.001469088 | 0.004028862 | 0.001476117 |
| hsa05332 | Graft-versus-host disease | 3/45 | 42/8146 | 0.001555826 | 0.004203053 | 0.001539938 |
| hsa04218 | Cellular senescence | 5/45 | 156/8146 | 0.001590893 | 0.004234583 | 0.00155149 |
| hsa04350 | TGF-beta signaling pathway | 4/45 | 94/8146 | 0.001725391 | 0.004526027 | 0.001658271 |
| hsa04630 | JAK-STAT signaling pathway | 5/45 | 162/8146 | 0.001879281 | 0.004859283 | 0.001780371 |
| hsa05215 | Prostate cancer | 4/45 | 97/8146 | 0.001936954 | 0.004937869 | 0.001809164 |
| hsa05225 | Hepatocellular carcinoma | 5/45 | 168/8146 | 0.002204388 | 0.005541587 | 0.002030358 |
| hsa04672 | Intestinal immune network for IgA production | 3/45 | 49/8146 | 0.002430543 | 0.006026416 | 0.002207992 |
| hsa04660 | T cell receptor signaling pathway | 4/45 | 104/8146 | 0.002499401 | 0.006113401 | 0.002239862 |
| hsa04062 | Chemokine signaling pathway | 5/45 | 192/8146 | 0.003926076 | 0.00947493 | 0.003471478 |
| hsa05022 | Pathways of neurodegeneration - multiple diseases | 8/45 | 476/8146 | 0.004069126 | 0.009690945 | 0.003550623 |
| hsa04919 | Thyroid hormone signaling pathway | 4/45 | 121/8146 | 0.00431409 | 0.010140912 | 0.003715484 |
| hsa05010 | Alzheimer disease | 7/45 | 384/8146 | 0.004710866 | 0.010931624 | 0.004005189 |
| hsa04142 | Lysosome | 4/45 | 132/8146 | 0.005871864 | 0.013343541 | 0.004888881 |
| hsa05221 | Acute myeloid leukemia | 3/45 | 67/8146 | 0.005897698 | 0.013343541 | 0.004888881 |
| hsa05211 | Renal cell carcinoma | 3/45 | 69/8146 | 0.006401091 | 0.014303672 | 0.005240659 |
| hsa04917 | Prolactin signaling pathway | 3/45 | 70/8146 | 0.006662129 | 0.014705432 | 0.005387858 |
| hsa04915 | Estrogen signaling pathway | 4/45 | 138/8146 | 0.006862188 | 0.014964531 | 0.005482788 |
| hsa04137 | Mitophagy - animal | 3/45 | 72/8146 | 0.007203085 | 0.015338334 | 0.005619744 |
| hsa05218 | Melanoma | 3/45 | 72/8146 | 0.007203085 | 0.015338334 | 0.005619744 |
| hsa04115 | p53 signaling pathway | 3/45 | 73/8146 | 0.007483075 | 0.015749263 | 0.005770303 |
| hsa05412 | Arrhythmogenic right ventricular cardiomyopathy | 3/45 | 77/8146 | 0.008667267 | 0.017831278 | 0.006533123 |
| hsa05171 | Coronavirus disease - COVID-19 | 5/45 | 232/8146 | 0.008669351 | 0.017831278 | 0.006533123 |
| hsa05226 | Gastric cancer | 4/45 | 149/8146 | 0.008954558 | 0.018210955 | 0.006672231 |
| hsa04662 | B cell receptor signaling pathway | 3/45 | 82/8146 | 0.010294382 | 0.020703146 | 0.007585334 |
| hsa04217 | Necroptosis | 4/45 | 159/8146 | 0.011186432 | 0.022249936 | 0.008152056 |
| hsa04012 | ErbB signaling pathway | 3/45 | 85/8146 | 0.011350327 | 0.022330535 | 0.008181586 |
| hsa04310 | Wnt signaling pathway | 4/45 | 167/8146 | 0.013210768 | 0.02571128 | 0.009420242 |
| hsa04658 | Th1 and Th2 cell differentiation | 3/45 | 92/8146 | 0.014050653 | 0.02705498 | 0.009912554 |
| hsa04141 | Protein processing in endoplasmic reticulum | 4/45 | 171/8146 | 0.014305479 | 0.027255703 | 0.009986096 |
| hsa05414 | Dilated cardiomyopathy | 3/45 | 96/8146 | 0.015744243 | 0.029684457 | 0.010875957 |
| hsa01522 | Endocrine resistance | 3/45 | 98/8146 | 0.016632488 | 0.030719187 | 0.011255067 |
| hsa05231 | Choline metabolism in cancer | 3/45 | 98/8146 | 0.016632488 | 0.030719187 | 0.011255067 |
| hsa04640 | Hematopoietic cell lineage | 3/45 | 99/8146 | 0.017087016 | 0.031239897 | 0.011445848 |
| hsa04360 | Axon guidance | 4/45 | 182/8146 | 0.017609126 | 0.031872518 | 0.011677631 |
| hsa05168 | Herpes simplex virus 1 infection | 7/45 | 495/8146 | 0.017811762 | 0.031920088 | 0.01169506 |
| hsa05330 | Allograft rejection | 2/45 | 38/8146 | 0.018492591 | 0.032815284 | 0.012023047 |
| hsa04064 | NF-kappa B signaling pathway | 3/45 | 104/8146 | 0.019464087 | 0.033874997 | 0.01241131 |
| hsa04625 | C-type lectin receptor signaling pathway | 3/45 | 104/8146 | 0.019464087 | 0.033874997 | 0.01241131 |
| hsa04931 | Insulin resistance | 3/45 | 108/8146 | 0.021491497 | 0.037047247 | 0.013573577 |
| hsa04940 | Type I diabetes mellitus | 2/45 | 43/8146 | 0.023344131 | 0.039861204 | 0.01460457 |
| hsa05415 | Diabetic cardiomyopathy | 4/45 | 203/8146 | 0.025161058 | 0.042562164 | 0.015594163 |
| hsa05203 | Viral carcinogenesis | 4/45 | 204/8146 | 0.025562722 | 0.042841229 | 0.015696408 |
| hsa04015 | Rap1 signaling pathway | 4/45 | 210/8146 | 0.028054662 | 0.046586182 | 0.017068505 |
| hsa05207 | Chemical carcinogenesis - receptor activation | 4/45 | 212/8146 | 0.0289167 | 0.047581116 | 0.017433035 |
